# Microfungi Associated with Peach Branch Diseases in China

**DOI:** 10.3390/jof10030217

**Published:** 2024-03-15

**Authors:** Ying Zhou, Ishara S. Manawasinghe, Zhizheng He, Wei Zhang, Mei Liu, Jinyan Song, Shifang Li, Zaifeng Fan, Jiye Yan

**Affiliations:** 1 State Key Laboratory for Biology of Plant Diseases and Insect Pests, Institute of Plant Protection, Chinese Academy of Agricultural Sciences, Beijing 100193, China; zhouying@ippbaafs.cn; 2 Beijing Key Laboratory of Environment Friendly Management on Fruit Diseases and Pests in North China, Key Laboratory of Environment Friendly Management on Fruit and Vegetable Pests in North China (Co-Construction by Ministry and Province), Ministry of Agriculture and Rural Affairs, Institute of Plant Protection, Beijing Academy of Agriculture and Forestry Sciences, Beijing 100097, China; 1873039722z@gmail.com (Z.H.); zhwei1125@163.com (W.Z.); liumeidmw@163.com (M.L.); 17866929429@163.com (J.S.); 3 College of Plant Protection, China Agricultural University, Beijing 100193, China; fanzf@cau.edu.cn; 4 Innovative Institute for Plant Health/Key Laboratory of Green Prevention and Control on Fruits and Vegetables in South China, Ministry of Agriculture and Rural Affairs, Zhongkai University of Agriculture and Engineering, Guangzhou 510225, China; ishara9017@gmail.com

**Keywords:** diversity, peach diseases, morphology, phylogenetic analyses, new species, new records

## Abstract

Peach (*Prunus persica* L.) is one of the most important and oldest stone fruits grown in China. Even though *P. persica* is one of the most commonly grown stone fruits in China, little is known about the biodiversity of microfungi associated with peach branch diseases. In the present study, samples were collected from a wide range of peach growing areas in China, and fungal pathogens associated with peach branch diseases were isolated. In total, 85 isolates were obtained and further classified into nine genera and 10 species. Most of the isolates belonged to *Botryosphaeriaceae* (46), including *Botryosphaeria*, *Diplodia*, *Neofusicoccum*, *Phaeobotryon*, and *Lasiodiplodia* species; *Ascochyta*, *Didymella*, and *Nothophoma* species representing *Didymellaceae* were also identified. Herein, we introduce *Ascochyta prunus* and *Lasiodiplodia pruni* as novel species. In addition, we report the first records of *Nothophoma pruni*, *Neofusicoccum occulatum*, and *Phaeobotryon rhois* on peach worldwide, and *Didymella glomerata*, *Nothophoma quercina*, and *Phaeoacremonium scolyti* are the first records from China. This research is the first comprehensive investigation to explore the microfungi associated with peach branch disease in China. Future studies are necessary to understand the pathogenicity and disease epidemiology of these identified species.

## 1. Introduction

Peach (*Prunus persica* L.) belongs to the family *Rosaceae* and is an important stone fruit that contains vitamins, flavonols, sugars, and catechins [1]. Peaches are rich in dietary fibres that provide health benefits [2]. They are consumed as fresh fruits and in processed foods such as jam and beverages. *Prunus* species were first domesticated and cultivated in northwestern China [3] and originated in China as long ago as 3300–2500 BC according to archaeological evidence [4]. According to the Food and Agricultural Organization 2019-United Nations (FAOSTAT), China is the top peach-producing country, with a production of 15.8 million tons in 2019, which accounted for 57% of the global production.

In China, the genetic diversity of peach is high, comprising more than 396 peach cultivars [5]. Peach cultivation areas in China are mainly located from the subtropical southern to the northern region and range from warm to cold and dry [6]. Peach trees are perennial plants that can grow up to 21 feet. Although the lifespan of peach plants is eight to ten years in orchards, it can reach 25 years [7]. Therefore, it is necessary to understand the biotic and abiotic factors affecting the vigour and yield of peach trees. Among various peach pests and diseases, fungal species are the dominant causative agents of diseases [8], such as brown rot caused by *Monilinia fructicola* [9]; gummosis caused by *Botryosphaeria dothidea* [10]; peach scab caused by *Venturia carpophila* [11]; peach leaf curl caused by *Taphrina deformans* [12]; and Phytophthora root and crown rot caused by *Phytophthora* spp. [13]. Worldwide, peach branch diseases including gummosis trunk, trunk canker, twig canker, twig spots, and shoot blight have been commonly observed, particularly in regions where stress factors are prevalent [14,15]. To date, there has been no comprehensive study on microfungi associated with peach branch diseases in China.

Even though peach fruits are among the most common stone fruits grown in China, few studies have been conducted to determine the diversity of microfungi associated with different peach cultivation regions in China. On this basis, the present study aimed to collect peach disease samples from a wide range of growing areas in China and to isolate and identify common fungal pathogens causing peach disease.

## 2. Materials and Methods

### 2.1. Sample Collection and Fungal Isolation

**Figure 1:**
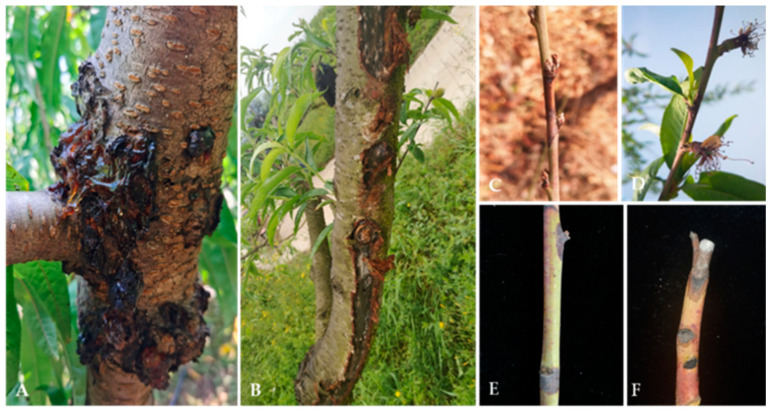
Disease symptoms on twigs, branches, and trunks of *Prunus persica* L. in the field. (**A**): Gummosis trunk; (**B**): trunk canker; (**C**,**D**): twig canker; (**E**): twig spots; and (**F**): shoot blight.

Diseased trunk, branch, and twig samples were collected from 20 peach orchards in 2020 and 2022 in Beijing, Hebei Province, Gansu Province, Liaoning Province, Guizhou Province, Sichuan Province, Yunnan Province, and Anhui Province in China. The disease samples were mainly collected from plants with typical dieback symptoms, such as gummosis, canker, twig canker spots, and shoot blight (Figure 1). The samples were cut into 0.5 × 0.5 cm pieces, surface sterilized for 1 min in 75% ethanol, rinsed for 2 min in distilled water, and blotted dry on sterilized filter paper. Then, the samples were transferred onto potato dextrose agar (PDA; 200 g potato, 20 g dextrose, and 20 g agar per L) plates and incubated at 25 °C to obtain pure cultures. Pure cultures were obtained via both single-tip isolation and single-spore isolation. The purified isolates were preserved on PDA slants at 4 °C [16].

### 2.2. Morphological and Cultural Characterization

The isolates were incubated on PDA at 25 °C or on malt extract agar (MEA; 30 g malt extract, 5 g mycological peptone, and 15 g agar/L), on oatmeal agar (OA; 40 g oatmeal and 5 g agar/L) under near-ultraviolet (UV) light (12 h light/12 h dark) when it was necessary, or on pine needle agar (PNA) [17] to induce sporulation. Colony diameters were measured after 5–7 days of incubation, and the culture characteristics were determined after 14 days [18]. Colony colours were recorded based on the colour charts of Rayner [19]. The micromorphological structures of mature conidiomata, conidia, and conidiogenous cells were studied on PDA, OA, and MEA [20,21]. Observations were conducted with an AxioCam 506 colour Imager Z2 photographic microscope (Carl Zeiss Microscopy, Oberkochen, Germany). Morphological features such as conidial length, width, and size were measured (at least 30/40 per isolate) with a ZEN Pro 2012 (Carl Zeiss Microscopy). The structure of the mature pycnidial wall was observed using microtome sections of 6–10 μm in thickness, which were prepared with a Leica CM 1950 freezing microtome (Leica Biosystems, Nussloch, Germany) and mounted in lactic acid [20,21,22]. All pure cultures obtained in this study were deposited in the culture collection of the Institute of Plant Protection, Beijing Academy of Agriculture and Forestry Sciences (JZB), China. All herbarium material including holotypes of novel species were deposited in the herbarium of the Institute of Plant Protection, Beijing Academy of Agriculture and Forestry Sciences (JZBH), China, as dry cultures.

### 2.3. DNA Extraction, PCR Amplification, and Sequencing

Fresh mycelia were scraped from the strains on PDA plates that were grown for seven days at 25 °C and collected in 1.5 mL centrifuge tubes. Genomic DNA was extracted using a TIANcombi DNA Lyse&Det PCR Kit (TIANGEN Biotech Co., Ltd., Beijing, China). Polymerase chain reaction (PCR) was carried out using selected genes and primers (Table 1 and Table 2). The 25 μL volume of each PCR mixture included 12.5 μL of 2× Taq PCR MasterMix (Beijing Bomede Gene Technology Co., Ltd., Beijing, China), 10.5 μL of ddH_2_O, 0.5 μL each of forward and reverse primer (Sangon Biotech, Shanghai, China), and 1 μL of DNA template. The thermal cycler conditions were as follows: initial denaturation for 3 min at 95 °C; 34 cycles of denaturation for 30 s at 95 °C, annealing for 30 s at 58 °C (the internal transcribed spacer region [ITS]), 56 °C (β-tubulin [*tub2*]; actin [*act*]), 54 °C (RNA polymerase II second largest subunit [*rpb2*]; partial translation elongation factor 1-alpha[*tef1*]), or 52 °C (28S large subunit of nuclear ribosomal RNA [LSU]); elongation at 72 °C; and a final extension for 10 min at 72 °C. The PCR products were assessed using agarose gel electrophoresis after staining with ethidium bromide and sequenced at Beijing Qingke Biotechnology Co., Ltd. (Beijing, China).

### 2.4. Phylogenetic Analyses

For all the isolates obtained in this study, sequence quality was assured by checking the chromatograms using BioEdit 7.0.9.0. All sequences were subjected to BLAST searches in the National Center for Biotechnology Information (NCBI) database using the Basic Local Alignment Search Tool (BLASTn) v. 2.15.0 (https://blast.ncbi.nlm.nih.gov/Blast.cgi) for preliminary identification of isolates. Based on the BLAST results, we identified our isolates as belonging to nine genera, *Ascochyta*, *Didymella*, *Nothophoma*, *Botryosphaeria*, *Diplodia*, *Neofusicoccum*, *Phaeobotryon*, *Lasiodiplodia*, and *Phaeoacremonium*. Reference sequences for phylogenetic analyses were retrieved from GenBank (https://www.ncbi.nlm.nih.gov/genbank/), following the recently updated taxonomic literature (Supplementary Table S1). The sequence dataset of each genus was aligned with MAFFT v. 7 (https://mafft.cbrc.jp/alignment/server/). BioEdit 7.0.9.0 was used to improve the alignment manually when necessary, such as trimming.

For the phylogenetic analysis of *Didymellaceae* species (*Ascochyta*, *Didymella*, and *Nothophoma*), gene regions were concatenated for the analyses in the order, ITS, LSU, *rpb2*, and *tub2* [23]. For *Botryosphaeriaceae* species (*Botryosphaeria*, *Diplodia*, *Lasiodiplodia*, and *Neofusicoccum*), gene regions were concatenated for the analyses in the order, ITS, *tef1*, and *tub2*, and for *Phaeobotryon* species, the combination of ITS, LSU, and *tef1* was used [24]. For *Phaeoacremonium* species, the *act* and *tub2* concatenated dataset was used [25]. Phylogenetic analyses were conducted using the maximum likelihood (ML) method implemented in RAxML [26,27], the maximum parsimony (MP) method in PAUP v. 4.0b10 [28], and Bayesian posterior probability analysis (BYPP) in MrBayes v.3.2.7a [29].

The ML analyses were performed with RAxML–HPC2 on XSEDE (8.2.12) [27,28] on the CIPRES Science Gateway platform [30] with 1000 nonparametric bootstrapping replicates, and the GTR + GAMMA was the nucleotide evolution model. Bayesian inference (BI) was performed in MrBayes v.3.2.7a [29] on the XSEDE tool. The evolution model was tested by using jModelTest2 on XSEDE in the CIPRES Gateway. MrBayes analyses were run for 1,000,000 generations, sampling the trees at every 100th generation. From the 10,000 trees obtained, the first 2000 representing the burn-in phase were discarded. The remaining 8000 trees were used to calculate posterior probabilities in the majority rule consensus tree.

In PAUP, tree stability was evaluated by 1000 bootstrap replications. Branches of zero length were collapsed, and all multiple most parsimonious trees were saved. Parameters, including tree–length (TL), consistency index (CI), retention index (RI), relative consistency index (RC), and homoplasy index (HI) were calculated. Differences between the trees inferred under different optimality criteria were evaluated using Kishino–Hasegawa tests (KHT). Phylogenetic trees were visualized in FigTree v1.4. The names of the isolates from the present study are marked in red in the trees. ML and MP bootstrap support values greater than 50% (BT) and Bayesian posterior probabilities (PPs) greater than 0.70 are given at the nodes.

**Table 1:** Gene regions and related primers used for molecular analysis.

Genes	Primers	Sequence (5′–3′)	References
ITS	ITS5 ITS4	GGAAGTAAAAGTCGTAACAAGG TCCTCCGCTTATTGATATGC	De Hoog and Gerrits van den Ende (1998) [31] White et al. (1990) [32]
LSU	LROR LR5	ACCCGCTGAACTTAAGC TCCTGAGGGAAACTTCG	Vilgalys and Hester (1990) [33] Rehner and Samuels (1994) [34]
*rpb2*	*RPB2*-5F *RPB2*-7cR	GAYGAYMGWGATCAYTTYGG CCCATRGCTTGYTTRCCCAT	Sung et al. (2007) [35] Liu et al. (1999) [36]
*tef1*	EF1-688F EF1-1251R	CGGTCACTTGATCTACAAGTGC CCTCGAACTCACCAGTACCG	Alves et al. (2008) [37]
	EF1-728F EF1-986R	CATCGAGAAGTTCGAGAAGG TACTTGAAGGAACCCTTACC	Carbone and Kohn (1999) [38]
*tub2*	Bt2a Bt2b	GGTAACCAAATCGGTGCTGCTTTC ACCCTCAGTGTAGTGACCCTTGGC	Glass and Donaldson (1995) [39]
	T1	AACATGCGTGAGATTGTAAGT	O’Donnell and Cigelnik (1997) [40]
	Btub2Fd Btub4Rd	GTBCACCTYCARACCGGYCARTG CCRGAYTGRCCRAARACRAAGTTGTC	Woudenberg et al. (2009) [41]
*act*	ACT-512F ACT-783R	ATGTGCAAGGCCGGTTTCGC TACGAGTCCTTCTGGCCCAT	Carbone and Kohn (1999) [38]

**Table 2:** Selected genes and primers for PCR amplification of each genus.

Family	Genera	ITS	LSU	*rpb2*	*tef1*	*act*	*tub2*
*Didymellaceae*	*Ascochyta*	ITS4/ITS5	LR0R/LR5	RPB2-5F2/ RPB2-7cR	*-*	-	Btub2Fd/ Btub4Rd
	*Didymella*	ITS4/ITS5	LR0R/LR5	RPB2-5F2/ RPB2-7cR	*-*	-	Btub2Fd/ Btub4Rd
	*Nothophoma*	ITS4/ITS5	LR0R/LR5	RPB2-5F2/ RPB2-7cR	*-*	-	Btub2Fd/ Btub4Rd
*Botryosphaeriaceae*	*Botryosphaeria*	ITS4/ITS5	-	-	EF1-728F/ EF1-986R	-	Bt2a/Bt2b
	*Diplodia*	ITS4/ITS5	-	-	EF1-728F/ EF1-986R	-	Bt2a/Bt2b
	*Neofusicoccum*	ITS4/ITS5	-	-	EF1-728F/ EF1-986R		Bt2a/Bt2b
	*Phaeobotryon*	ITS4/ITS5	LROR/LR5	-	EF1-728F/ EF1-986R	-	-
	*Lasiodiplodia*	ITS4/ITS5	-	-	EF1-688F/ EF1-1251R	-	T1/Bt2b
*Togniniaceae*	*Phaeoacremonium*	-	-	-	-	ACT-512F/ ACT-783R	Bt2a /Bt2b

## 3. Results

In the present study, a total of 85 isolates were obtained. These isolates were further identified as belonging to three families and nine genera: *Ascochyta* (eight isolates), *Didymella* (six isolates), *Nothophoma* (22 isolates), *Botryosphaeria* (33 isolates), *Diplodia* (four isolates), *Neofusicoccum* (three isolates), *Phaeobotryon* (three isolates), *Lasiodiplodia* (three isolates), and *Phaeoacremonium* (three isolates). For all identified taxa, updated phylogenetic trees, species descriptions, and illustrations are given. All identified taxa are listed based on the current outline of fungi [23].

### Phylogenetic Analysis and Morphological Characterization

**Dothideomycetes** O.E. Erikss. & Winka.

For the currently accepted treatment of Dothideomycetes, we followed Hongsanan et al. [24].

***Didymellaceae*** Gruyter, Aveskamp & Verkley, Mycological Research 113 (4): 516 (2009).

*Didymellaceae* is a species-rich family that exhibits a global distribution pattern [42]. Furthermore, forty-four genera and more than 5400 species are accepted in *Didymellaceae* [23]. In the present study, we isolated and identified species belonging to three *Didymellaceae* genera, namely, *Ascochyta* (eight isolates), *Didymella* (six isolates), and *Nothophoma* (22 isolates).

***Ascochyta*** Lib., Plantae Cryptogamicae quas in Arduenna collegit M.A. Libert Fasc. 1: 8 (1830).

**Figure 2:**
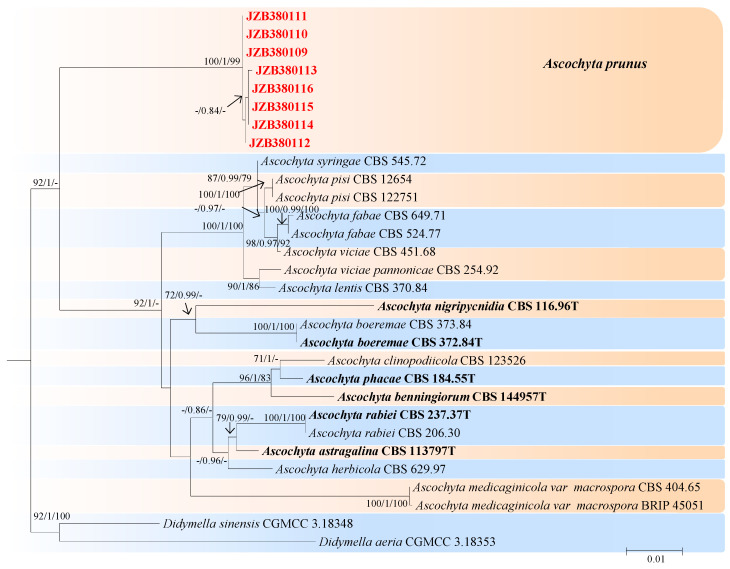
Maximum likelihood (ML) phylogram reconstructed from the combined sequences of LSU, ITS, *rpb2*, and *tub2* of *Ascochyta* species. Bootstrap support values for ML and maximum parsimony (MP) greater than 50% and Bayesian posterior probabilities greater than 0.70 are indicated above the branches as ML BS/PP/MP BS. The scale bar represents the expected number of changes per site. The tree is rooted with *Didymella sinensis* (CGMCC 3.18348) and *Didymella aeria* (CGMCC 3.18353). The novel species proposed are indicated in red font, and the type specimens are indicated in bold.

*Ascochyta* is a prominent genus that encompasses not only pathogens but also saprophytic and endophytic fungi that exist on a wide range of substrates [43,44,45,46,47]. The combined dataset of LSU, ITS, *rpb2*, and *tub2* ingroup isolates from 15 species consisted of 2276 characters (891 for LSU, 490 for ITS, 596 for *rpb2*, and 299 for *tub2*), including alignment gaps. Based on the results of jModel test for BI, TrN + I was determined to be the best model for the LSU dataset, TIM2ef + I was determined to be the best model for the ITS dataset, TIM3 + I + G was determined to be the best model for the *rpb2* dataset, and TIM3 + G was determined to be the best model for the *tub2* dataset. *Didymella aeria* (CGMCC 3.18353) and *Didymella sinensis* (CGMCC 3.18348) were used as outgroup taxa. The best-scoring ML tree with a final likelihood value of −5834.327678 is shown in Figure 2. The matrix had 281 distinct alignment patterns, with 12.84% undetermined characters or gaps. The parameters for the model of the combined dataset were as follows: estimated base frequencies, A = 0.239085, C = 0.240356, G = 0.275806, T = 0.244754; substitution rates, AC = 1.072413, AG = 4.489167, AT = 1.459809, CG = 0.739866, CT = 13.212760, and GT = 1.000000; and gamma distribution shape parameter α = 0.020000. According to the results of the multilocus phylogenetic analysis, eight isolates from *P. persica* in this study were clustered into an independent branch with 92% bootstrap support and 1.0 BYPP (Figure 2).

**Figure 3:**
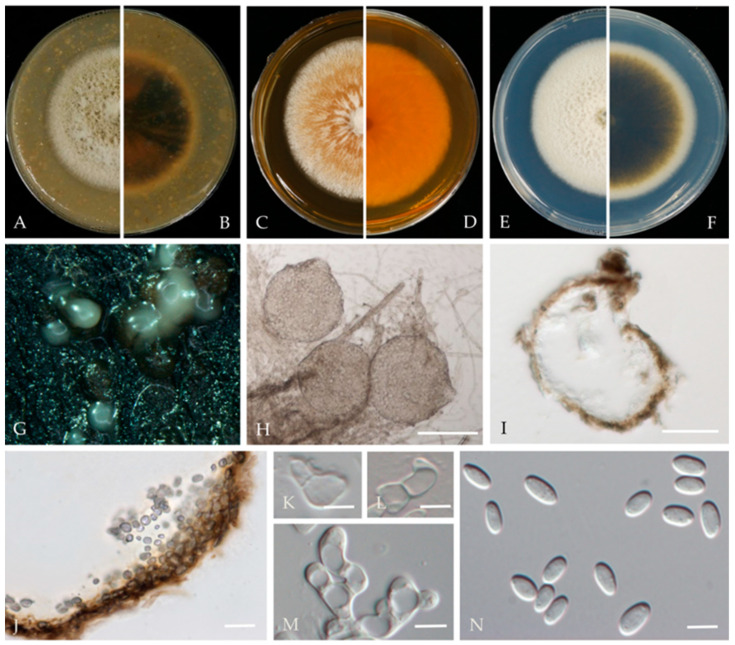
***Ascochyta prunus*** (JZB380109, Ex-type) (**A**,**B**) Colony on OA (front and reverse) (**C**,**D**) Colony on MEA (front and reverse) (**E**,**F**) Colony on PDA (front and reverse) (**G**) Pycnidia forming on OA. (**H**) Pycnidium. (**I**) Section through the pycnidium. (**J**) Section of the pycnidial wall. (**K**–**M**) Conidiogenous cells. (**N**) Conidia. Scale bars: (**H**) = 100 μm; (**I**) = 50 μm; and (**J**–**N**) = 5 μm.

***Ascochyta prunus*** Y. Zhou, W. Zhang & J.Y. Yan, sp. nov., (Figure 3).

MycoBank number: MB851694.

Etymology—The name refers to the host genus, *Prunus*.

Holotype—JZBH380109.

Associated with twig canker and branch canker in *Prunus persica.* **Sexual morph**: not observed. **Asexual morph**: *Conidiomata* pycnidial, solitary or aggregated; (sub) globose or flask-shaped; glabrous; semi-immersed in or superficial on the agar; ostiolate, 104–225 (−30) × 95–185 (−20) μm. *Ostiole* single. The *pycnidial wall* is pseudoparenchymatous and composed of oblong to isodiametric cells, 2–4 layers, and 6–11 μm thick, with 2–3 layers pigmented. *Conidiogenous cells* 4–8 × 3–6 μm (av. = 6.1 × 4.9 μm, n = 30), phialidic, hyaline, smooth, (sub) globose, ampulliform to lageniform, without pigmented layers. *Conidia* 3.9–6.5 × 2.2–3.5 μm (av. = 5.5 × 2.9 μm, n = 50), greatly variable in shape and size, oblong, ovoid, or broad ellipsoidal, smooth and thin-walled, aseptate.

**Culture characteristics**—Colonies on OA were 80–81 mm in diameter after 7 days, margin regular, covered by floccose aerial mycelia, dense, white; reverse black. Colonies grown on MEA had an 84–85 mm diameter after 7 days; margin regular, aerial mycelia sparse, flattened, light grey to white, with some radial line near the centre, reverse concolourous. Colonies on PDA were similar to those on OA but somewhat slower growing, with a 74–76 mm diameter after 7 days, covered by floccose aerial mycelia that were whiter and denser than those on OA, reverse olivaceous (Figure 3).

**Material examined**—Changping and Miyun Districts, Beijing municipality, China, from twig canker and branch canker of *Prunus persica*, May 2020 and May 2021. Y Zhou. W Zhang, (holotype JZBH380109 as dry culture, paratype JZBH380110-JZBH380116 as dry cultures); ex type living cultures JZB380109, ex paratype JZB380110-JZB380116.

**Notes**—In the phylogenetic analysis of the present study, eight isolates from *Prunus* developed a distinct lineage from other known *Ascochyta* species with 100% ML, 99% MP bootstrap, and 1.00 BYPP values. Phylogenetically, our isolates showed close affinity to *A. pisi*, but they can be distinguished by their conidial length, whereas our isolates developed smaller conidia (3.9–6.5 µm) than *A. pisi* (7–16 µm, CBS 122785; 10–16 µm, CBS 122751) [21,48]. The nucleotide differences between JZB380109 and *A. pisi* (CBS 126.54) are LSU: 1.13% (1/880 bp), ITS: 2.46% (12/487 bp), *rpb2*: 13.88% (126/886 bp), and *tub2*: 4.50% (15/333 bp). Based on molecular phylogeny and morphology, herein we introduce isolates from this study as *Ascochyta pruni*, a new species from China.

***Didymella*** Sacc., Michelia 2 (6): 57 (1880).

*Didymella* was established by Saccardo in 1880, with the description of *Didymella exigua* [42]. These species are plant pathogens and saprobes on a wide range of hosts [42]. In the present study, six isolates were identified as belonging to *Didymella.* The combined dataset of LSU, ITS, *rpb2*, and *tub2* with 13 species as ingroup consisted of 2239 characters (854 for LSU, 488 for ITS, 597 for *rpb2*, and 300 for *tub2*, including alignment gaps). TrN + I was determined to be the best model for the LSU dataset, TIM2ef + I was determined to be the best model for the ITS dataset, TrN + G was determined to be the best model for the *rpb2* dataset, and TrN + I was determined to be the best model for the *tub2* dataset. *Ascochyta boeremae* (CBS 373.84) and *Ascochyta fabae* (CBS 524.77) were used as outgroup taxa.

**Figure 4:**
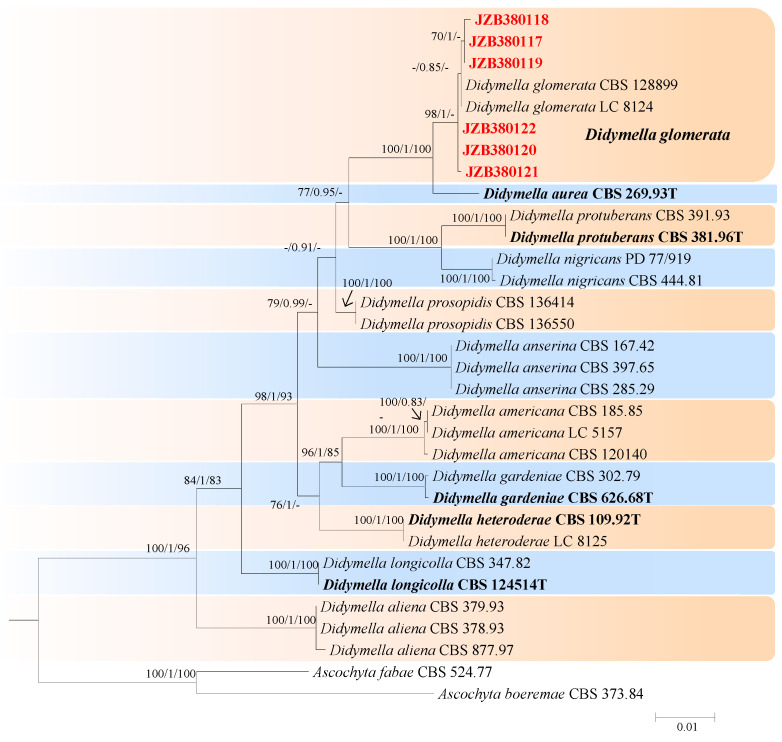
Maximum likelihood (ML) phylogram reconstructed from the combined sequences of LSU, ITS, *rpb2*, and *tub2* of *Didymella* isolates. Bootstrap support values for ML and maximum parsimony (MP) greater than 50% and Bayesian posterior probabilities greater than 0.70 are indicated above the branches as ML BS/PP/MP BS. The scale bar represents the expected number of changes per site. The tree is rooted with *Ascochyta fabae* (CBS 524.77) and *Ascochyta boeremae* (CBS 373.84). Isolates from this study are marked in red, and the type specimens are indicated in bold.

The best-scoring ML tree with a final likelihood value of −5647.047275 is shown in Figure 4. The matrix had 242 distinct alignment patterns, with 1.88% undetermined characters or gaps. The parameters for the model of the combined dataset were as follows: estimated base frequencies: A = 0.236386, C = 0.243959, G = 0.279575, and T = 0.240080; substitution rates: AC = 1.269086, AG = 4.594322, AT = 1.024640, CG = 0.692657, CT = 14.061872, and GT = 1.000000; and gamma distribution shape parameter α = 0.020000. According to the results of the phylogenetic analyses of this study, our strains were clustered together with *Didymella glomerata*, with 98% ML and 1.00 BYPP values (Figure 4).

**Figure 5:**
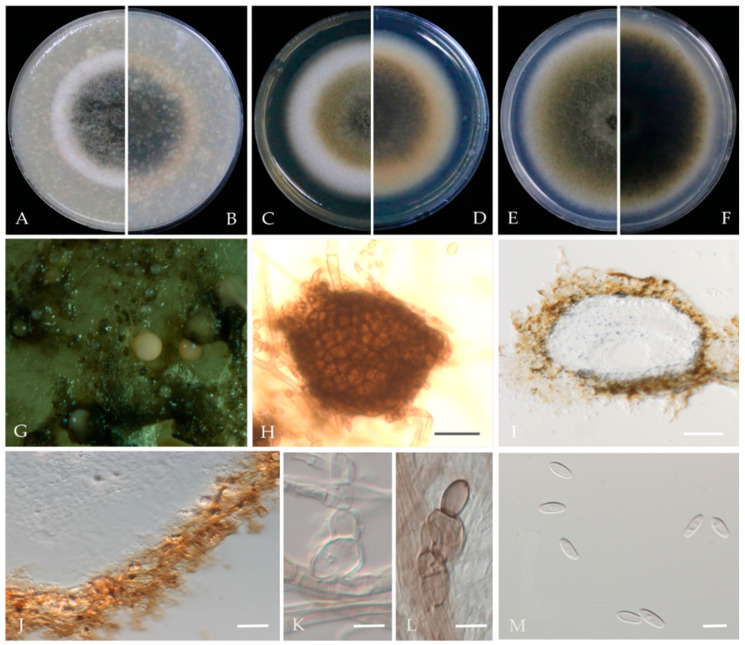
***Didymella glomerata*** (JZB380117) (**A**,**B**) Colony on OA (front and reverse) (**C**,**D**) Colony on MEA (front and reverse) (**E**,**F**) Colony on PDA (front and reverse) (**G**) Pycnidia forming on OA. (**H**) Pycnidium. (**I**) Section through the pycnidium. (**J**) Section of the pycnidial wall. (**K**,**L**) Conidiogenous cells. (**M**) Conidia. Scale bars: (**H**,**I**)= 50 μm; (**J**) = 10 μm; and (**K**–**M**) = 5 μm.

***Didymella glomerata*** (Corda) Qian Chen & L. Cai, Stud. Mycol. 82: 176 (2015) (Figure 5).

MycoBank number: MB814105;

≡ *Phoma glomerata* (Corda) Wollenw. & Hochapfel, Z. Parasitenk. 8: 592. 1936.

≡ *Peyronellaea glomerata* (Corda) Goid. ex Togliani, Ann. Sperim. Agrar. III 6: 93. 1952.

Associated with twig canker in *Prunus persica.* **Sexual morph**: not observed. **Asexual morph:** *Conidiomata pycnidial*, solitary or aggregated, globose or subglobose, 100–240 × 80–220 μm diameter, glabrous, semi-immersed in or superficial on the agar, papillate, with 1 ostiole. *Pycnidial wall*, pseudoparenchymatous and composed of isodiametric cells, 3–7 layers, outer layers pigmented. *Conidiogenous cells* 5–10 × 5–9 μm (av. = 8.7 × 7.6 μm, n = 30), phialidic, hyaline, smooth, mostly ampulliform, sometimes (sub) globose. *Conidia* 5–8 × 2.5–3.5 μm (av. = 7.1 × 3.6 μm, n = 50), greatly variable in shape and size, oblong, ovoid or obovate, smooth- and thin-walled, aseptate, partially guttulate.

**Culture characteristics**—Colonies on OA, 55–57 mm in diameter after 7 days, margin regular, smoky grey to grey olivaceous, white near the margin, covered by fluffy, dense, white to grey aerial mycelia; reverse concolourous and white near the margin. Colonies on MEA, 68–69 mm in diameter after 7 days, margin regular, covered by floccose, white and greenish olivaceous aerial mycelia, reverse concolourous, white near the margin. Colonies on PDA, 76–79 mm in diameter after 7 days, similar to those on MEA but somewhat faster growing and sparser, reverse concolourous, white margin narrower than those on OA and MEA.

**Material examined**—Changping and Miyun Districts, Beijing municipality, China, from the twig canker of *Prunus persica*, Aug. 2021. Y Zhou.; Living cultures JZB380117–JZB380122.

**Notes**—Six isolates from twig spot and gummosis trunk of peach (*Prunus persica* L.) in this study were phylogenetically related to *Didymella glomerata* (Figure 4). *Didymella glomerata*, known to cause diseases in dicots and conifers, is generally found in the rhizosphere flora but has recently been identified as a cause of stem canker in peach trees, damping off and root necrosis in fennel, and stem rot in coriander [49,50,51,52]. This is the first report of this fungus on *Prunus persica* in China.

***Nothophoma*** Qian Chen & L. Cai, Stud. Mycol. 82: 212 (2015).

This genus was described by Chen et al. [21] and typified with *Nothophoma infossa*. There are 23 accepted species in this genus (Index Fungorum 2023). For the taxonomic treatments of this genus, we followed Keirnan et al. [53]. The combined dataset of LSU, ITS, *rpb2*, and *tub2* ingroup isolates from seven species consisted of 2228 characters (848 for LSU, 485 for ITS, 596 for *rpb2*, and *299* for *tub2*, including alignment gaps). TrN was determined to be the best model for the LSU dataset, K80 was determined to be the best model for the ITS dataset, TIM3 + G was the best model for *rpb2*, and TrN + G was the best model for *tub2*. *Didymella protuberans* (CBS 391.93) and *Didymella protuberans* (CBS 381.96) were used as outgroup taxa.

**Figure 6:**
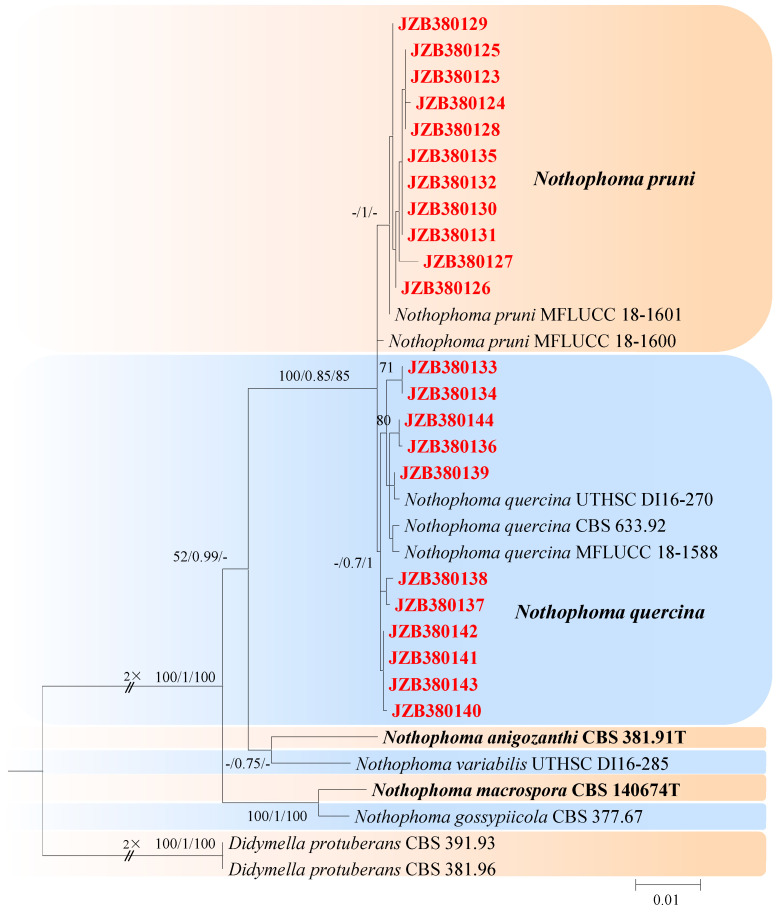
Maximum likelihood (ML) phylogram reconstructed from the combined sequences of LSU, ITS, *rpb2*, and *tub2* of *Nothophoma* isolates. Bootstrap support values for ML and maximum parsimony (MP) greater than 50% and Bayesian posterior probabilities greater than 0.70 are indicated above the branches as ML BS/PP/MP BS. The scale bar represents the expected number of changes per site. The tree is rooted with *Didymella protuberans* (CBS 391.93) and *Didymella protuberans* (CBS 381.96). Isolates from this study are marked in red, and the type specimens are indicated in bold.

The best-scoring ML tree with a final likelihood value of −4754.240240 is given in Figure 6. The matrix had 180 distinct alignment patterns, with 6.46% undetermined characters or gaps. The parameters for the model of the combined dataset were as follows: estimated base frequencies, A = 0.237873, C = 0.242414, G = 0.279069, and T = 0.240643; substitution rates, AC = 1.232725 and AG = 3.271098; AT = 1.025485; CG = 0.670080; CT = 11.394298; and GT = 1.000000; and gamma distribution shape parameter α = 0.020000 (Figure 6).

***Nothophoma pruni*** Chethana, J.Y. Yan, X.H. Li & K.D. Hyde, Mycosphere 10 (1): 520 (2019) Figure 7.

MycoBank number: MB828518.

Associated with twig spot and gummosis trunk of *Prunus persica.* **Sexual morph**: not observed. **Asexual morph:** *Conidiomata pycnidial*, solitary or aggregated on agar, globose to irregularly shaped, black, and ostiolate, measuring 63–240 × 60–230 μm, single and conspicuous. *Pycnidial wall* pale brown, pseudoparenchymatous, composed of isodiametric cells, 3–6 layers, 1–2 outer layers slightly pigmented. *Conidiogenous cells* are phialidic, hyaline, doliiform to ampulliform, and variable in size. *Conidia* 4–7 × 3–4.7 μm (av. = 5.9 × 3.8 μm, n = 50), variable in shape and size, cylindrical to obovoid or oblong, thin-walled, smooth, aseptate, hyaline.

**Culture characteristics**—Colonies on OA 57–60 mm in diameter after 7 days with regular margins. Aerial mycelium white, floccose to woolly. Immersed mycelium grey–green olivaceous to deep olivaceous near the colony centre and grey near the margin; reverse concolourous. Colonies on MEA 36–41 mm in diameter after 7 days, margin regular. Aerial mycelia covering the whole colony, compact, white to pale grey; reverse concentric circles of different colours, orange to yellow. Colonies on PDA, 60–66 mm in diameter after 7 days, aerial mycelium sparse, white to grey–green; reverse deep brown, grey near the margin.

**Material examined**—Pinggu, Changping, and Haidian districts, Beijing municipality, China, from twig spot and gummosis trunk of *Prunus persica*. Mar and Jul 2021. Y Zhou, DL Ma, Y Li.; living cultures JZB380123, JZB380125-JZB380132, and JZB380135. Apr 2021. Y Zhou, DL Ma, Y Li; living culture JZB380124.

**Notes**—In the present study, 11 isolates from twig spots and gummosis trunks of peach were phylogenetically closely related to *Nothophoma pruni* (Figure 6). *Nothophoma pruni* has been reported as saprobic on diseased leaves of *Prunus avium* [54]. This is the first report of this fungus on the host *Prunus persica* worldwide.

***Nothophoma quercina*** Qian Chen & L. Cai. in Qian Chen and L. Cai, Stud. Mycology 82: 213 (2015) Figure 8.

MycoBank number: MB814086.

Associated with shoot blight and gummosis in *Prunus persica*. **Sexual morph**: not observed. **Asexual morph:** *Conidiomata pycnidial*, solitary or aggregated on agar, globose, or peroblate to suboblate, measuring 130–320 × 120–270 μm with a single, conspicuous, nonpapillate ostiole. *Pycnidial wall* pale brown, pseudoparenchymatous, composed of isodiametric cells, 3–5 layers, and 1–2 outer layers that are slightly pigmented. *Conidiogenous cells*, 6–10 × 4–8 μm (av. = 8.3 × 6.3 μm, n = 30), phialidic, hyaline, smooth, doliiform to ampulliform, variable in size. *Conidia* 4–6 × 3–5 μm (av. = 5.0 × 3.9 μm, n = 50), variable in shape and size, subglobose to oval or obtuse, thin-walled, smooth, aseptate, initially hyaline, light brown when mature.

**Culture characteristics**—Colonies on OA were 61–69 mm in diameter after 7 days with regular margins. Aerial mycelium white, floccose to woolly. Immersed mycelium grey–green olivaceous to light olivaceous near the colony centre and white near the margin; reverse concolourous. Colonies on MEA were 55–75 mm in diameter after 7 days, margins regular. Aerial mycelia covering the whole colony were compact, white to pale grey, with some radially furrowed zones; reverse concentric circles of different colours, orange to yellow and light yellow near the margin. Colonies on PDA were 65–68 mm in diameter after 7 days, margins regular, covered by floccose, white and greenish olivaceous aerial mycelia, reverse concolourous, light green near the margin.

**Material examined**—Pinggu and Haidian districts, Beijing municipality, China, from shoot blight and gummosis trunk of *Prunus persica*. May, Jul 2020 and Mar, Jul 2021. Y. Zhou, DL. Ma, and Y. Li, living cultures JZB380133, JZB380134, and JZB380136-JZB380144.

**Notes**—In this study, 11 isolates obtained from shoot blight and gummosis trunk of peach (*Prunus persica*) were phylogenetically closely related to *Nothophoma quercina* (Figure 6). Morphologically, our isolates share the same characteristics as given in the type species description [21]. *Nothophoma quercina* has been reported as the main pathogen causing branch blight [55,56]. This is the first report of this fungus infecting the host *Prunus persica* in China.

**Dothidiomycetes families *incertae sedis.***

For taxonomic treatments, we followed Hongsanan et al. [24].

***Botryosphaeriaceae*** Theiss. & Syd. Annales Mycologici 16 (1–2): 16 (1918).

*Botryosphaeriaceae* includes diverse pathogenic members that are classified as plant opportunistic fungal pathogens [57,58]. Species of *Botryosphaeriaceae* cause gummosis and shoot blight disease in peach [59,60,61,62,63]. These species are also important pathogens of grapevines and are associated with a variety of diseases [64]. Additionally, more than 20 species of *Botryosphaeriaceae* have been reported to cause *Botryosphaeria* dieback [65]. For taxonomic treatments, we followed Hongsanan et al. [24] and Wu et al. [66].

***Botryosphaeria*** Ces. & De Not., Comment. Soc. Crittog. Ital. 1 (4): 211 (1863) [MB#635].

For the taxonomic treatment of this genus, we followed Zhang et al. [67]. The combined dataset of ITS, *tef1*, and *tub2* ingroup isolates from nine species consisted of 1436 characters (611 for ITS, 362 for *tef1*, and 463 for *tub2*, including alignment gaps). TrN + G was determined to be the best model for the ITS dataset, TPM2uf + I was the best model for the *tef1* dataset, and TIM3 + G was the best model for the *tub2* dataset. *Diplodia corticola* (CBS 112546) and *Diplodia corticola* (CBS 112549) were used as outgroup taxa.

**Figure 9:**
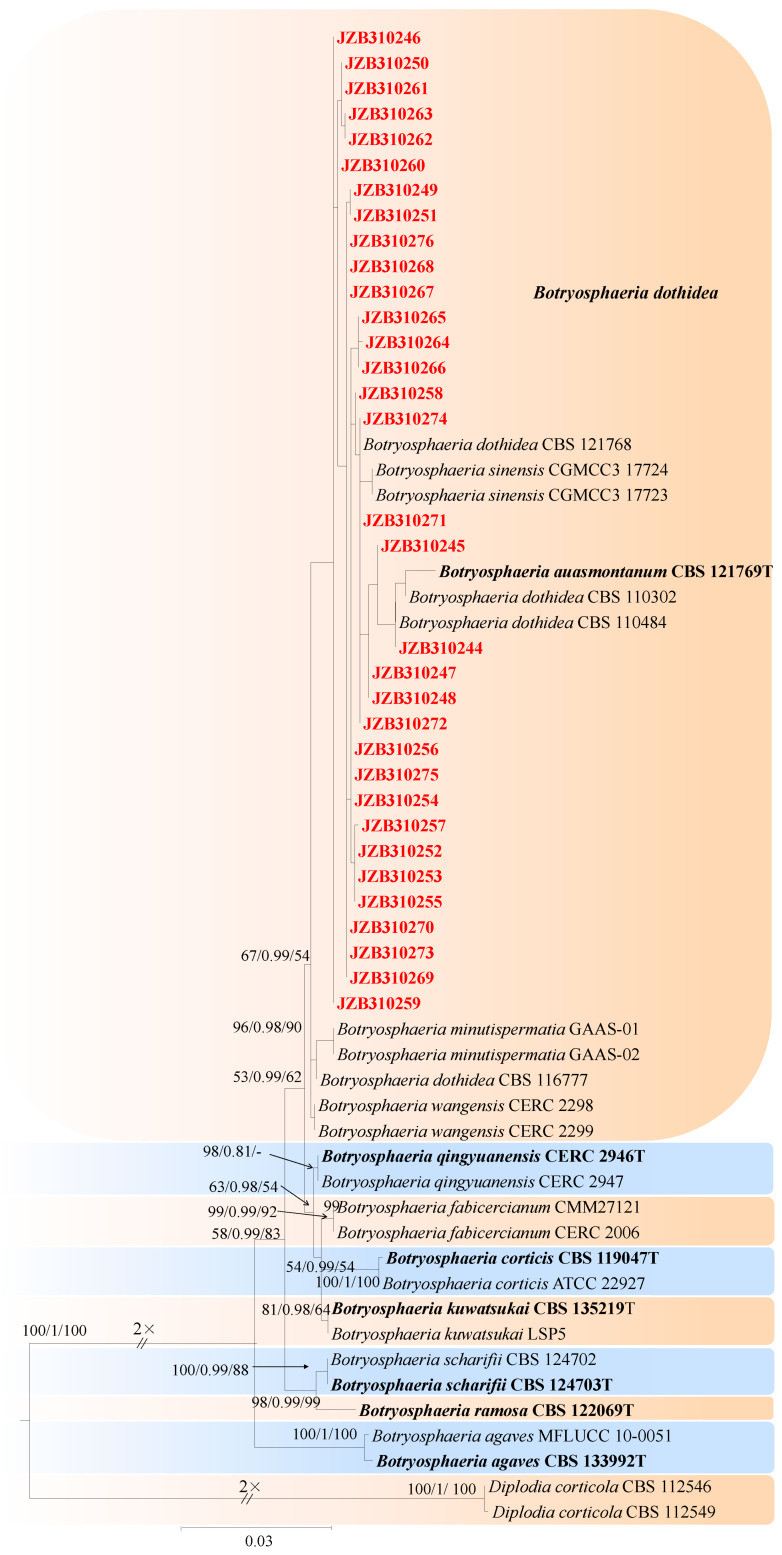
Maximum likelihood (ML) phylogram reconstructed from the combined ITS, *tef1*, and *tub2* sequences of *Botryosphaeria* isolates. Bootstrap support values for ML and maximum parsimony (MP) greater than 50% and Bayesian posterior probabilities greater than 0.70 are indicated above the branches as ML BS/BPP/MP BS. The scale bar represents the expected number of changes per site. The tree is rooted with *Diplodia corticola* (CBS 112546) and *Diplodia corticola* (CBS 112549). Isolates from this study are marked in red. and the type specimens are indicated in bold.

The best-scoring ML tree with a final likelihood value of −3605.559999 is shown in Figure 9. The matrix had 327 distinct alignment patterns, 16.87% of which were undetermined characters or gaps. The parameters for the model of the combined dataset were as follows: estimated base frequencies, A = 0.216286, C = 0.301284, G = 0.257502, and T = 0.224928; substitution rates, AC = 1.420115, AG = 2.115530, AT = 1.012019, CG = 1.284585, CT = 3.876955, and GT = 1.000000; and gamma distribution shape parameter α = 0.319888 (Figure 9).

**Figure 10:**
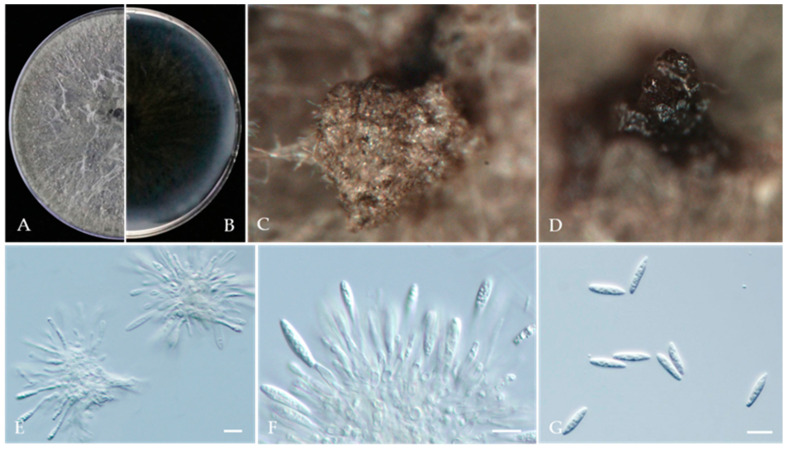
*Botryosphaeria dothidea* (JZB310251). (**A**,**B**) Colony on PDA (front and reverse); (**C**,**D**): Appearance of conidiomata on PNA; (**E**,**F**) Conidiogenous cells; (**G**) Conidia; Scale bars: (**E**–**G**) = 20 μm.

***Botryosphaeria dothidea*** (Moug.) Ces. & De Not., Comm. Soc. crittog. Ital. 1(fasc. 4): 212 (1863) (Figure 10).

MycoBank number: MB183247;

*Basionym: Sphaeria dothidea* Moug., *In*: Fries, Syst. Mycol. (Lundae) 2(2): 423. 1823.

= *Botryosphaeria berengeriana* De Not., Sfer. Ital. 82. 1863 [1864].

= *Fusicoccum aesculi* Corda, *In*: Sturm, Deutschl. Fl., Abth. 3, 2: 111. 1829.

= *Sphaeria coronillae* Desm., Annls Sci. Nat., Bot., sér. 2 13: 188. 1840.

≡ *Macrophoma coronillae* (Desm.) Höhn., Ber. Deutsch. Bot. Ges. 28:479. 1910.

≡ *Macrophomopsis coronillae* (Desm.) Petr., Annls mycol. 22(1/2): 108. 1924.

≡ *Dothiorella coronillae* (Desm.) Petr., Sydowia 16(1–6): 188. 1963.

≡ *Fusicoccum coronillae* (Desm.) Vanev. & Aa, In: van der Aa & Vanev, A Revision of the Species Described in Phyllosticta (Utrecht): 192. 2002.

= *Phyllosticta divergens* Sacc., Malpighia 5: 274. 1891.

Associated with Prunus persica branch canker. **Sexual morph**: not observed. **Asexual morph**: *Conidiomata* pycnidial, solitary, globose to ovoid, dark brown to black, embedded in needle tissue, semi-immersed to superficial, with a central ostiole. *Conidiogenous cells* holoblastic, discrete, hyaline, cylindrical to lageniform, phialidic with periclinal thickening, 11–18 × 2–4 μm (av. = 16.1 × 3.1 μm, n = 30). Paraphyses not observed. *Conidia* hyaline, thin-walled, smooth with granular contents, aseptate, narrowly or irregularly fusoid, base subtruncate to bluntly rounded, apex subobtuse, 18–28 × 4–7 μm (av. = 23.9 × 5.8 μm, n = 50; L/W = 4.1).

**Culture characteristics**—Colonies on PDA had fluffy aerial mycelia with irregular margins, with appressed moderately dense mycelial mats that were initially white and then smoky grey to dark olivaceous, covering the dish after 5 days at 25 °C in the dark.

**Material examined**—Pinggu, Changping, and Haidian districts, Beijing municipality, China, from branch canker of *Prunus persica*, May 2020, May 2021, and July 2021. Y Zhou & Z.Z Zhi; living cultures JZB310240-JZB310243, JZB310245-JZB310258, and JZB310267-JZB310276; Hebei Province, Qianghuangdao City, Changli County, from branch canker of *Prunus persica*, September 2021. Y Zhou, living cultures JZB310261, JZB310262, and JZB310263; Guizhou Province, Guiyang City, Kaiyang County, from branch canker of *Prunus persica*, Apr. 2021. Y Zhou & Y Li, living cultures JZB310244, JZB310254, JZB310255, JZB310264, JZB310265, and JZB310266; and Sichuan Province, Mianyang City, from gummosis trunk of *Prunus persica*, September 2021. Y Zhou & JH Jiang, living cultures JZB310259, JZB310260.

**Note**—In the phylogenetic analysis of the present study, 15 isolates from branch canker and gummosis trunk of peach from four provinces in China were clustered together with *Botryosphaeria dothidea* (Figure 9). Further, these isolates were morphologically similar to those given in the type species description. *Botryosphaeria dothidea* is an opportunistic pathogen with a wide host range [68]. It has been reported to cause shoot blight [63], and it is also related to gummosis-causing agents [62].

***Diplodia*** Fr., Ann. Sci. Nat., Bot. Sér. 2, 1: 302 (1834).

For the taxonomic treatment of this genus, we followed Zhang et al. [67]. The *combined* dataset of ITS, *tef1*, and *tub2* ingroup isolates of 13 species consisted of 1261 characters (540 for ITS, 300 for *tef1*, and 421 for *tub2*, including alignment gaps). TPM3 + I + G was determined to be the best model for ITS, TrN + G was determined to be the best model for *tef1*, and TrN + I + G was the best model for the *tub2* dataset. *Lasiodiplodia theobromae* (CBS 164.96) was used as the outgroup taxon.

**Figure 11:**
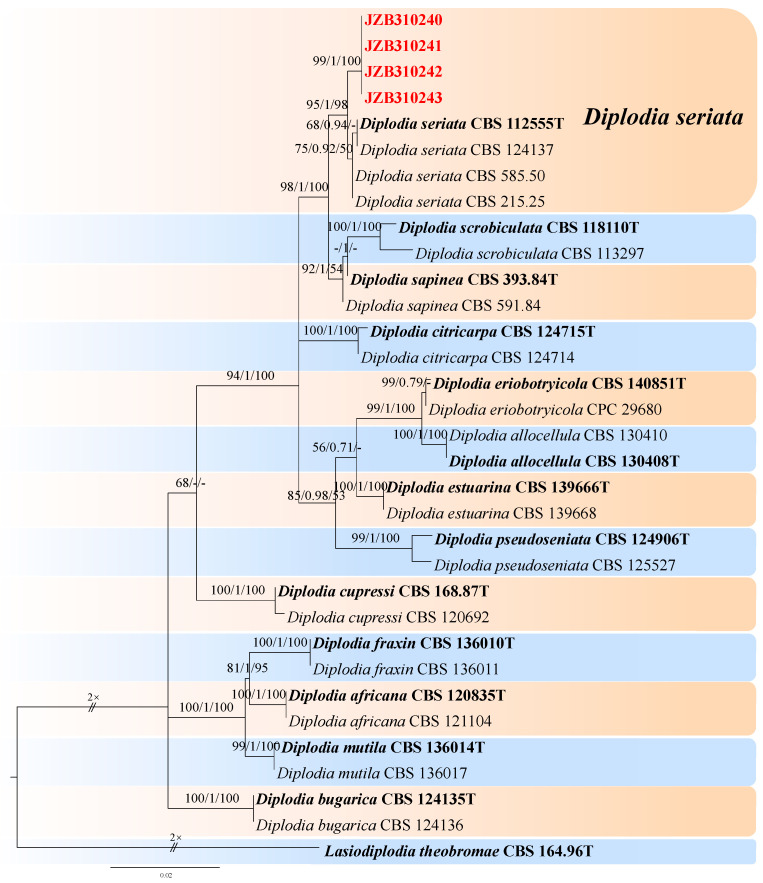
Maximum likelihood (ML) phylogram reconstructed from the combined ITS, *tef1*, and *tub2* sequences of *Diplodia* isolates. Bootstrap support values for ML and maximum parsimony (MP) greater than 50% and Bayesian posterior probabilities greater than 0.70 are indicated above the branches as ML BS/BPP/MP BS. The scale bar represents the expected number of changes per site. The tree is rooted with *Lasiodiplodia theobromae* (CBS 164.96). Isolates from this study are marked in red, and the type specimens are indicated in bold.

The best-scoring ML tree with a final likelihood value of −3415.055016 is given in Figure 11. The matrix had 270 distinct alignment patterns, with 7.54% undetermined characters or gaps. The parameters for the model of the combined dataset were as follows: estimated base frequencies, A = 0.200294, C = 0.314841, G = 0.255463, and T = 0.229402; substitution rates, AC = 0.879909, AG = 2.534595, AT = 1.049732, CG = 1.056783, CT = 5.084602, and GT = 1.000000; and gamma distribution shape parameter α = 0.104584 (Figure 11).

***Diplodia seriata*** De Not., Mém. R. Accad. Sci. Torino, Ser. 2 7: 26 (1845) Figure 12.

MycoBank number: MB180468.

Associated with twig spots on *Prunus persica*. **Sexual morph**: not observed. **Asexual morph**: *Conidiomata pycnidial*, solitary, globose to ovoid, dark brown to black, embedded, semi-immersed to superficial. *Conidiogenous cells* hyaline, smooth, thin-walled, and discrete, producing a single conidia at the tip, proliferating internally and giving rise to periclinal thickening or proliferating concurrently, forming 2–3 annellations, 8–15 × 3–6 μm (av. = 11.7 × 4.6 μm, n = 30). *Conidia* hyaline, thin-walled, smooth, aseptate, ovoid, apex subobtuse, becoming brown when mature, 19–28 × 9–12 μm (av. = 23.2 × 10.4 μm, n = 50; L/W = 2.2).

**Culture characteristics**—Colonies on PDA had fluffy aerial mycelia with irregular margins, appressed moderately dense mycelial mats and smoky grey to dark olivaceous, covering the dish after 5 days at 25 °C in the dark.

**Material examined**—Pinggu district, Beijing municipality, China, from twig spots on *Prunus persica*, April 2020. Y Zhou (living culture JZB310240-JZB31043).

**Notes**—In the present study, we examined the morphology and phylogeny of samples of peach twig spots from China and identified these isolates as *Diplodia seriata* (Figure 11). *Diplodia seriata* (syn. *B. obtusa*) has been reported in many countries and is recognized as an important pathogen of stone, pome, and soft fruit trees, causing cankers, leaf spots, and black fruit rot [69,70,71,72].

***Neofusicoccum*** Crous, Slippers & A.J.L. Phillips, Stud. Mycol. 55: 247 (2006).

*Neofusicoccum* was introduced by Crous et al. [73] as a species that is morphologically similar to but phylogenetically distinct from *Botryosphaeria* and thus could no longer be included in that genus. For the taxonomic treatment of this genus, we followed Zhang et al. [67]. The *combined* dataset of ITS, *tef1*, and *tub2* included 24 ingroup isolates from 11 species and consisted of 1406 characters 542 for ITS, 441 for *tef1*, and 423 for *tub2*, including alignment gaps. TIM1 + I was determined to be the best model for the ITS dataset, HKY + G was determined to be the best model for the *tef1* dataset, and TrN + G was determined to be the best model for the *tub2* dataset. *Botryosphaeria dothidea* (CBS 115476) was used as the outgroup taxon.

**Figure 13:**
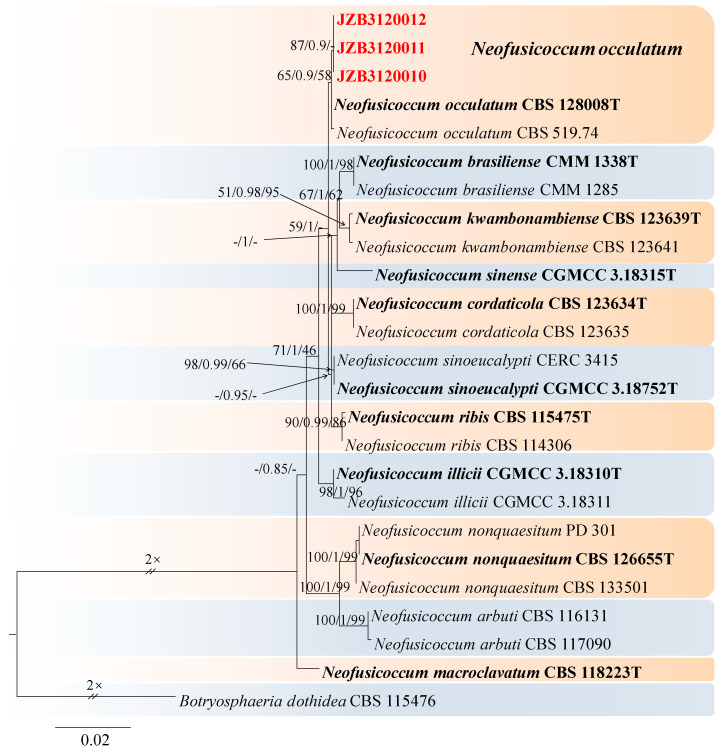
Maximum likelihood (ML) phylogram reconstructed from the combined ITS, *tef1*, and *tub2* sequences of *Neofusicoccum* isolates. Bootstrap support values for ML and maximum parsimony (MP) greater than 50% and Bayesian posterior probabilities greater than 0.70 are indicated above the branches as ML BS/BPP/MP BS. The scale bar represents the expected number of changes per site. The tree is rooted with *Botryosphaeria dothidea* (CBS 115476). Isolates from this study are marked in red, and the type specimens are indicated in bold.

The best-scoring ML tree with a final likelihood value of −2311.055412 is given in Figure 13. The matrix had 101 distinct alignment patterns, with 8.20% undetermined characters or gaps. The parameters for the model of the combined dataset were as follows: estimated base frequencies, A = 0.204892, C = 0.316100, G = 0.266872, and T = 0.212136; substitution rates, AC = 0.804456, AG = 7.315164, AT = 3.072031, CG = 1.357888, CT = 9.437879, and GT = 1.000000; and gamma distribution shape parameter α = 1.010866 (Figure 13).

**Figure 14:**
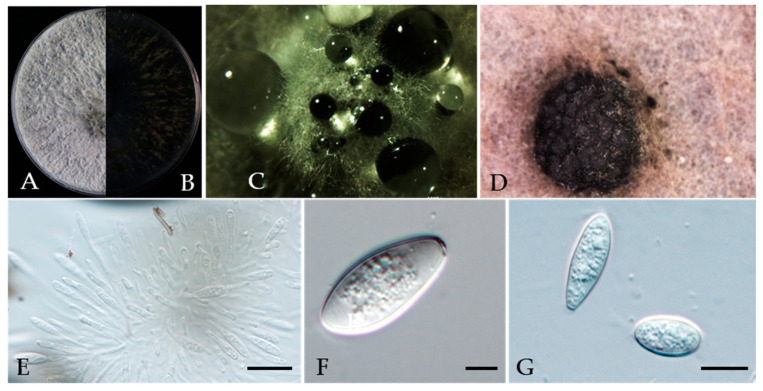
***Neofusicoccum occulatum*** (JZB3120010) (**A**,**B**) Colony on PDA (front and reverse); (**C**,**D**) appearance of conidiomata on PDA; (**E**) conidia developing on conidiogenous cells; (**F**,**G**) conidia; Scale bars: (**E**) = 20 µm; (**F**) = 5 µm; and (**G**) = 10 µm.

***Neofusicoccum occulatum*** Sakalidis & T. Burgess, Molecular Phylogenetics and Evolution 60 (3): 333–344 (2010) (Figure 14).

MycoBank number: MB518777.

Associated with twig canker on *Prunus persica*. **Sexual morph**: not observed. **Asexual morph**: *Conidiomata* pycnidial, produced on PDA, solitary, globose to ovoid, dark brown to black, 924–2566 µm. *Conidiogenous cells* discrete, hyaline, cylindrical to lageniform, 7–25 × 1.6–3.4 μm (av. = 12.8 × 2.5 μm, n = 30). *Conidia*, hyaline, smooth, fusiform to ellipsoidal with an obtuse apex, thin-walled, septate, 14–21 × 6–10 μm (av. = 18 × 7 μm, n = 50; L/W = 2.5).

**Culture characteristics**—Colony on PDA superficial, grey, fluffy, reverse dark brown to black, and colonies covering the 90 mm diameter Petri dish were incubated for 5 days in the dark at 25 °C.

**Material examined**—Changping district, Beijing municipality, China, from the twig canker of *Prunus persica*, Aug. 2021. Y Zhou (living cultures JZB3600010- JZB3600012).

**Notes**—In the phylogenetic analysis of the present study, three isolates obtained from *Prunus* clustered together with the *Neofusicoccum occulatum* type species (CBS128008). Morphologically, our isolates have similar characteristics to the *Ne. occulatum* type species [74], thus we identified our isolates as *Ne. occulatum. Neofusicoccum occulatum* was reported as the pathogen causing shoot blight in *Platycladus orientalis* [74]. In the present study, we presented the morphology and phylogeny of peach twig canker samples from China and identified these isolates as *Neofusicoccum occulatum* (Figure 13 and Figure 14).

***Phaeobotryon*** Theiss. & Syd., Annales Mycologici 13 (3–4): 664 (1915).

*Phaeobotryon* was introduced by Theiss. and Syd. to accommodate *Dothidae cercidis* as *Phaeobotryon cercidis* and the species which are phylogenetically and morphologically distinguished from the other genera in *Botryosphaeriaceae* [57,75]. For the taxonomic treatment of this genus, we followed Zhang et al. [67]. The *combined* dataset of ITS, LSU, and *tef1* from 21 ingroup isolates of seven species consisted of 1272 characters (449 for ITS, 558 for LSU, and 265 for *tef1*, including alignment gaps). TIM1ef + I was determined to be the best model for the ITS dataset, TrN + I for the LSU dataset, and HKY + G was determined to be the best model for the *tef1* dataset. *Barriopsis iraniana* (CBS 124698) was used as the outgroup taxon.

**Figure 15:**
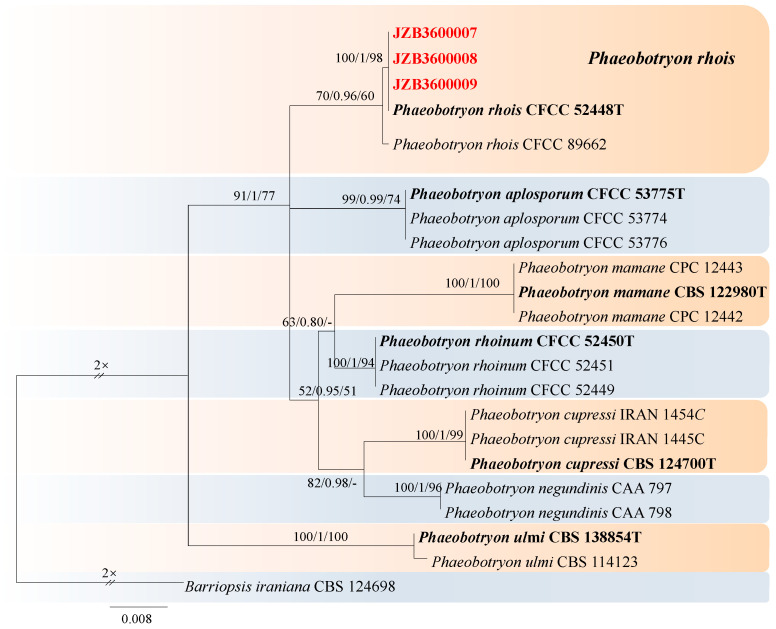
Maximum likelihood (ML) phylogram reconstructed from the combined sequences of ITS, LSU, and *tef1* of *Phaeobotryon* isolates. Bootstrap support values for ML and maximum parsimony (MP) greater than 50% and Bayesian posterior probabilities greater than 0.70 are indicated above the branches as ML BS/BPP/MP BS. The scale bar represents the expected number of changes per site. The tree is rooted with *Barriopsis iraniana* CBS 124698. Isolates from this study are marked in red, and the type specimens are indicated in bold.

The best-scoring ML tree with a final likelihood value of −2724.623212 is given in Figure 15. The matrix had 144 distinct alignment patterns, 19.13% of which were undetermined characters or gaps. The parameters for the model of the combined dataset were as follows: estimated base frequencies, A = 0.225969, C = 0.265270, G = 0.277126, and T = 0.231634; substitution rates, AC = 0.866717, AG = 2.192592, AT = 0.532373, CG = 0.670797, CT = 5.651745, and GT = 1.000000; and gamma distribution shape parameter α = 0.836999 (Figure 15).

**Figure 16:**
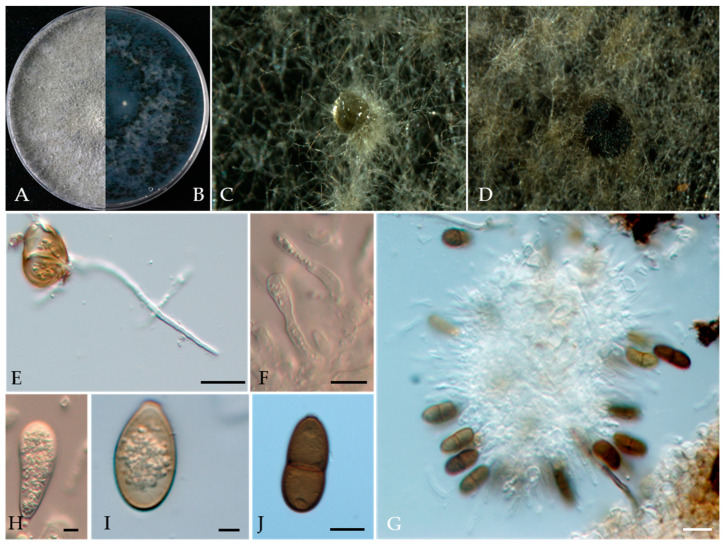
***Phaeobotryon rhois*** (JZB3600007) (**A**,**B**) Colony on PDA (front and reverse); (**C**,**D**) Appearance of conidiomata on PDA; (**E**) Germinating conidia; (**F**,**G**) Conidia developing on conidiogenous cells; (**H**–**J**) Conidia; Scale bars: (**E**) = 20 µm; (**F**) = 10 µm; (**G**) = 20 µm; (**H**,**I**) = 5 µm; and (**J**) = 10 µm.

***Phaeobotryon rhois*** C.M. Tian, X.L. Fan & K.D. Hyde, Phytotaxa 205(2): 95 (2015) (Figure 16).

MycoBank number: MB 811599; Facesoffungi number: FoF 00596.

Associated with twig canker of *Prunus persica* **Sexual morph**: not observed. **Asexual morph**: *Conidiomata* pycnidial, produced on PDA, solitary, globose to ovoid, dark brown to black, 274.73–1155 µm. *Conidiogenous cells* discrete, hyaline, cylindrical to lageniform, 6–18 × 3–7 μm (av. = 13.3 × 4.3 μm, n = 30). *Conidia* ellipsoid to oblong or subcylindrical or obovoid, smooth to verruculose, moderately thick-walled, guttulate, ends rounded, initial hyaline, aseptate, becoming brown, 1-septate when mature, 18–29 × 10–16 μm (av. = 25 × 13 μm, n = 50, L/W = 1.9).

**Culture characteristics**—The colonies were originally white and produced dark green to black pigments after they had been incubated for 7–10 days. The texture was felty with an appressed mycelial mat and fluffy aerial mycelia near the centre, with regular edges. Colonies reached the 90 mm diameter of a Petri dish after 5 days in the dark at 25 °C.

**Material examined**—Pinggu district, Beijing municipality, China, from the twig canker of *Prunus persica*, Aug. 2021. Y Zhou, DL Ma, and ZZ He, living cultures JZB3600007-JZB3600009.

**Notes**—In the present study, we examined the morphology and phylogeny of peach twig canker samples from China and identified these isolates as *Phaeobotryon rhois* (Figure 16)*. Phaeobotryon rhois* is known to cause canker and dieback disease in *Rhus typhina* in China [76]. This is the first report of *P. rhois* being associated with twig canker disease in peach.

***Lasiodiplodia*** Ellis & Everh., Bot. Gaz. 21: 92 (1896).

*Lasiodiplodia* species are cosmopolitan and have an extensive host and geographical range. They are pathogenic on economically important fruit crops [57]. For the taxonomic treatment of this genus, we followed Zhang et al. [67] and Xia et al. [77]. The combined ITS, *tef1*, and *tub2* dataset of 79 ingroup strains from 43 species consisted of 1158 characters, (415 for ITS, 332 for *tef1*, and 411 for *tub2*, including alignment gaps). TVM + I was determined to be the best model for the ITS dataset, HKY + I + G was determined to be the best model for the TEF dataset, and TrN + I was determined to be the best model for the *tub2* dataset. *Diplodia seriata* (CBS 112555) and *Diplodia mutila* (CMW 7060) were used as the outgroup taxon.

**Figure 17:**
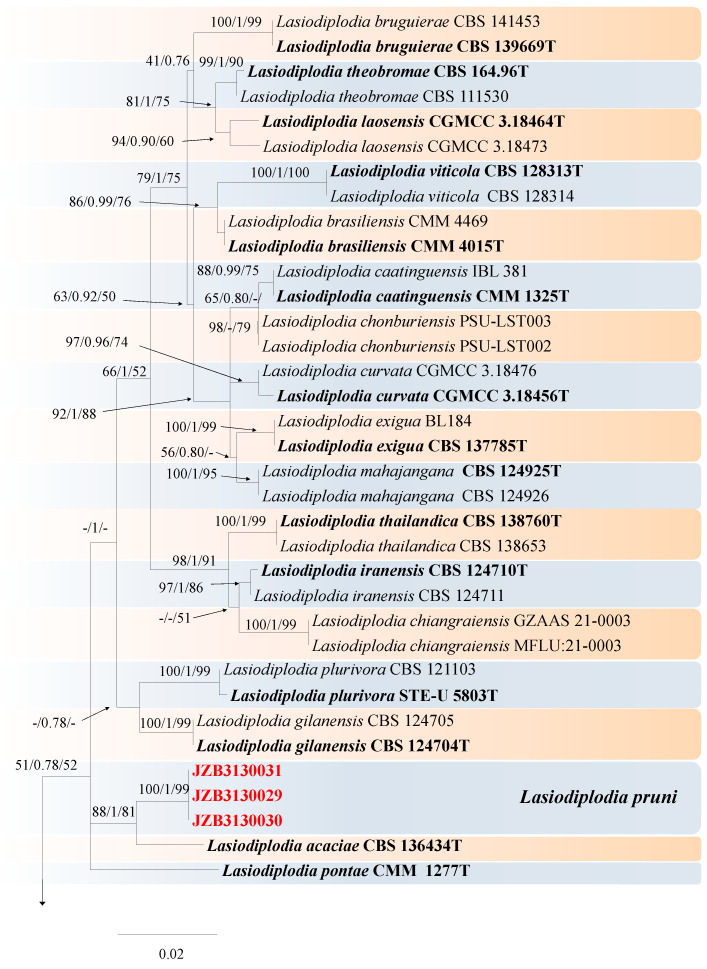

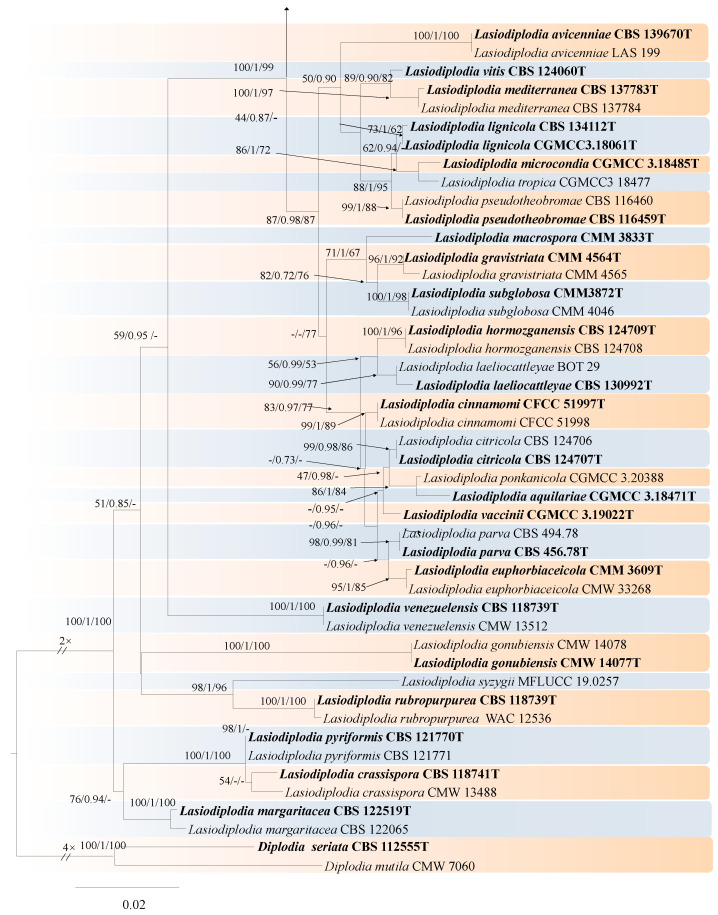
Maximum likelihood (ML) phylogram reconstructed from the combined sequences of ITS, *tef1*, and *tub2* of *Lasiodiplodia* isolates. Bootstrap support values for ML and maximum parsimony (MP) greater than 50% and Bayesian posterior probabilities greater than 0.70 are indicated above the branches as ML BS/BPP/MP BS. The scale bar represents the expected number of changes per site. The tree is rooted with *Diplodia seriata* (CBS 112555) and *Diplodia mutila* (CMW 7060). Isolates from this study are marked in red, and the type specimens are indicated in bold.

The best-scoring ML tree with a final likelihood value of −5119.098465 is given in Figure 17. The matrix had 357 distinct alignment patterns, 13.73% of which were unde-termined characters or gaps. The parameters for the model of the combined dataset were as follows: estimated base frequencies, A = 0.209134, C = 0.307908, G = 0.255624, and T = 0.227335; substitution rates, AC = 1.006140, AG = 3.800814, AT = 1.344431, CG = 0.991903, CT = 5.144516, and GT = 1.000000; and gamma distribution shape parameter α = 0.798124 (Figure 17).

**Figure 18:**
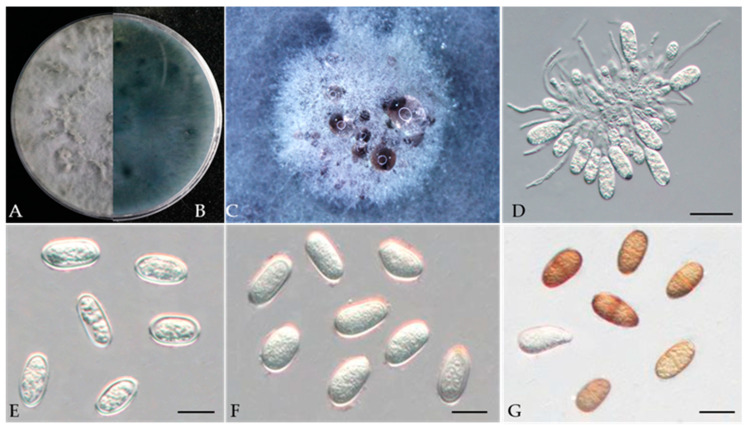
***Lasiodiplodia pruni*** (JZB3130029, ex type) (**A**,**B**) Colony on PDA (front and reverse); (**C**) Appearance of conidiomata on PDA; (**D**) Conidia developing on conidiogenous cells; (**E**,**F**) Young, hyaline conidia; (**G**) Mature, brown, 1-septate conidia; Scale bars: (**D**) = 20 µm and (**E**–**G**) = 10 µm.

***Lasiodiplodia pruni*** Y. Zhou, W. Zhang & J.Y. Yan, sp. nov., (Figure 18).

MycoBank number: MB 852445.

Etymology—The name refers to the host genus, *Prunus*.

Holotype-JZBH3130029.

Associated with gummosis trunk in *Prunus persica.* **Sexual morph**: not observed. **Asexual morph**: *Conidiomata* pycnidial produced on PDA, superficial or rarely semi-immersed, black, solitary, globose to subglobose with a central ostiole, with or without papilla. *Conidiogenous cells* 10–23 × 3–6.8 μm (av. = 17.7 × 6.6 μm, n =30) hyaline, smooth, cylindrical, holoblastic, *Conidia* initially hyaline, thick-walled, had a wall 0.6–1.1 μm thick, ellipsoid to ovoid with a rounded or slightly tapered apex, 12.8–16.4 × 6.4–9.2 μm (av. = 14.7 × 7.8 μm, n = 50, L/W = 1.8), then turn brown with a median septum and longitudinal striations when mature, 11.9–17.1 × 6.6–8.7 μm (av. 14.2 × 7.6 μm, n = 50, L/W = 1.87).

**Culture characteristics**—Colonies on PDA had fluffy aerial mycelia, with an appressed mycelial mat that was sparse to moderately dense, a few cottony aerial mycelia reaching the lid of the Petri dish, irregular margins, and smoky grey; additionally, colonies reached the 90 mm diameter Petri dish after 3 days in the dark at 25 °C.

**Materials examined**—China, Anhui Province, Liuan City, gummosis trunk of peach (*Prunus persica* L.). Sep. 2023, Y Zhou, (holotype JZBH3130029 as dry culture and JZBH3130030 and JZBH3130031 as dry cultures); ex-type living culture JZB3130029, living cultures JZBH3130030 and JZB3130031.

**Notes**—In the phylogenetic analysis, three isolates from the present study developed a particular sister relationship with *L. acacia* with 88% ML, 81% MP bootstrap, and 1.00 BYPP values. Morphologically our isolates are different from *L. acaciae*, by conidial sizes where our isolates develop smaller conidia (av. = 14.2 × 7.6 μm) than *L. acaciae* (CBS 136434) (av. = 27.3 × 12.9 μm) [67]. The nucleotide differences between JZB3130029 and *L. acaciae* (CBS 136434) were ITS: 0.47% (2/420 bp), *tef1*: 3.15% (14/444 bp), and *tub2*: 0% (0/447 bp). Based on the phylogenetic analysis and morphology, we introduce our isolates as *Lasiodiplodia pruni*, a novel species from China.

***Togniniaceae*** Réblová, L. Mostert, W. Gams & Crous, Stud. Mycol. 50(2): 540 (2004).

***Phaeoacremonium*** W. Gams, Crous & M.J. Wingf., Mycologia 88 (5): 789 (1996). [MB#27679].

*Phaeoacremonium* comprises common pathogens that cause stem and branch diseases in a wide range of woody hosts [78]. In 2021, *P. minimum* was first reported as a pathogen causing esca disease in China [79]. In this study, we followed Ye et al. [23] for taxonomic treatments.

**Figure 19:**
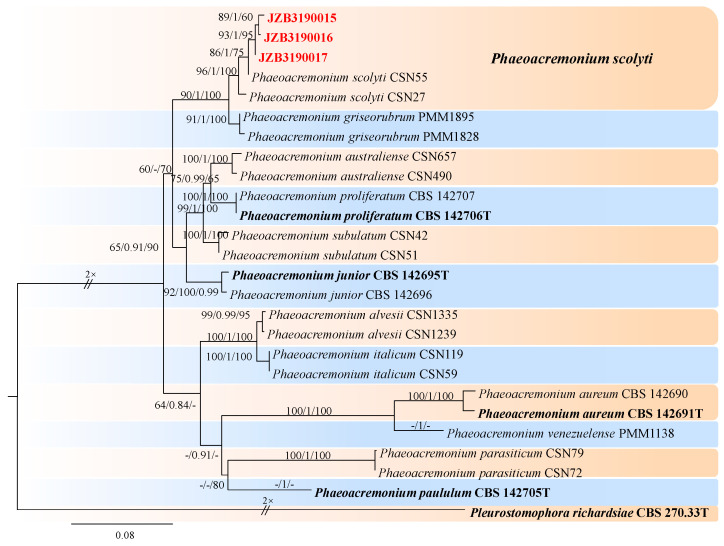
Maximum likelihood (ML) phylogram reconstructed from the combined sequences of *act* and *tub2* from *Phaeoacremonium* isolates. Bootstrap support values for ML and maximum parsimony (MP) greater than 50% and Bayesian posterior probabilities greater than 0.70 are indicated above the branches as ML BS/BPP/MP BS. The scale bar represents the expected number of changes per site. The tree is rooted with *Pleurostomophora richardsiae* (CBS 270.33). Isolates from this study are marked in red, and the type specimens are indicated in bold.

The *combined* dataset of *act* and *tub2* contained 25 ingroup isolates from 12 species and consisted of 880 characters (259 for *act* and 621 for *tub2*, including alignment gaps). HKY + G was determined to be the best model for the act dataset, and TPM2uf + G was the best model for the *tub2* dataset. *Pleurostomophora richardsiae* (CBS 270.33) was used as the outgroup taxon. The best-scoring ML tree with a final likelihood value of −4698.584463 is given in Figure 19. The matrix had 391 distinct alignment patterns, with 6.55% undetermined characters or gaps. The parameters for the model of the combined dataset were as follows: estimated base frequencies, A = 0.204162, C = 0.311249, G = 0.238989, and T = 0.245600; substitution rates, AC = 1.158313, AG = 4.407917, AT = 1.364022, CG = 0.995655, CT = 4.976526, and GT = 1.000000; and gamma distribution shape parameter α = 0.505328 (Figure 19).

**Figure 20:**
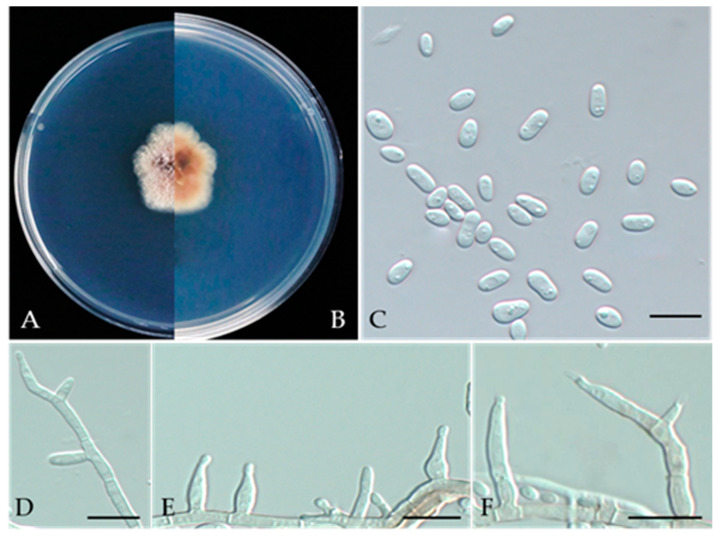
***Phaeoacremonium scolyti*** (JZB3190015) (**A**,**B**) Colony on PDA (front and reverse); (**C**) Conidia on PDA; (**D**) Type I phialides; (**E**) Type II phialides; (**F**) Type III phialides; Scale bars: (**C**–**F**) = 10 µm.

***Phaeoacremonium scolyti*** L. Mostert, Summerb. & Crous, J. Clin. Microbiol. 43 (4): 1763 (2005) (Figure 20).

MycoBank number: MB357048.

Associated with gummosis trunk of *Prunus persica*. **Sexual morph**: not observed. **Asexual morph**: Mycelia consist of branched septate hyphae. *Conidiophores* mostly short and usually unbranched, subcylindrical to navicular. Type I phialides cylindrical and occasionally swollen at 2.4–7.1 × 1.0–1.9 μm (av. 5.29 × 1.38 μm, n = 30). Type II phialides predominant, elongate-ampulliform, attenuated or constricted at the base, or navicular tapering towards the apex, 7–14 × 1.5–2.7 μm (av. 9.28 × 1.89 μm, n = 30); type III phialides subcylindrical, subulate to elongate-ampulliform, 13–25 × 1.5–2.5 μm (av. 17.46 × 1.94 μm, n = 30), tapering gradually to the apex. *Conidia* oblong-ellipsoidal or obovoid, occasionally reniform or allantoid, 2.0–4.8 × 1.1–3.0 μm (av. 3.20 × 1.92 μm, n = 50, L/W=1.67).

**Culture characteristics**—colonies on PDA were flat, felty to woolly, with irregular edges at a radius of 10–12 mm after 9 days at 25 °C; after 9 days, the cells became pinkish white to hyaline/translucent.

**Materials examined**—China, Liaoning Province, Huludao City, gummosis trunk of *Prunus persica* L., Sep. 2022, Y Zhou, living cultures JZB3190015-JZB3190017.

**Notes**—Three isolates obtained from gummosis trunk disease of peach (*Prunus persica*) were phylogenetically closely related to *P*. *scolyti. P*. *scolyti* is also known as *V. vinifera* in South Africa [80,81]. This fungus had the broadest host range and was found on the *Prunus* species sampled [82].

## 4. Discussion

China is the world’s largest producer of peach. The peach orchards in China are commonly affected by cankers, leaf spots, and fruit rot diseases. However, compared to fruit rot and other fruit diseases, trunk disease is usually disregarded even though trunk diseases directly affect the lifespan of the plant. In the present study, we focused on trunk, branch, and twig-inhabiting fungi, and 85 isolates were obtained from diseased peach trunks, branches, and twigs. The isolates were identified as 10 fungal species belonging to nine genera using phenotypic characteristics and a multilocus phylogeny. Among them, two species were identified as new, three species were reported on peach in China for the first time, and four species were reported on peach for the first time worldwide. Among these isolates, *Didymellaceae* and *Botryosphaeriaceae* were the most common taxa, and *Didymellaceae* (42.4%) and *Botryosphaeriaceae* (54.1%) accounted for more than 90% of the total isolates.

*Didymellaceae* is a species-rich family that features a diverse range of fungi that showcase global distribution patterns. Additionally, many of these fungi are economically important plant pathogens [42]. We isolated and identified species belonging to three *Didymellaceae* genera, and *Nothophoma* was the most frequently isolated genus. Species belonging to this genus are pathogens, endophytes, and saprobes on economically important crops and forest trees [54]. *Nothophoma quercina* (syn. *Phoma fungicola*) was the main pathogen causing branch blight [55,56]. This fungus produces abundant pycnidia on plant residues, which can subsequently become the primary source of infection. Under favourable conditions, such as cloudy and cool weather (moisture above 70% and temperature between 20 and 25 °C), the fungus can immediately asexually reproduce and kill the host [83]. *Nothophoma pruni* has been reported to be a saprobe on diseased leaves of *Prunus avium* [54]. In this study, we first isolated *Nothophoma pruni* from twig spot and gummosis trunk samples of peach from around the world. *N*. *quercina* was first isolated from shoot blight and gummosis trunk samples of peach in China.

*Ascochyta* was introduced by Libert in 1830, with *A. pisi* described as a type species [84]. Some of the species have been reported as plant pathogens; *Ascochyta syringae* causes Ascochyta blight of lilac (*Syringa vulgaris*) in America, Australia, and Europe [46], and it has been isolated mostly from soil [47]. In the present study, *A*. *prunus* was isolated from twig canker and branch canker samples of *P. persica* as a novel species. *Ascochyta prunus* was distinguished by its conidial length compared to *A. pisi* (CBS 122785) and *A. pisi* (CBS 122751).

*Didymella glomerata* (former name *Phoma glomerata*) is a globally distributed soil fungus that has been isolated from various plants (more than 100 host plant genera). Generally, it is considered a secondary invasive or opportunistic pathogen [18]. *Didymella glomerata* is associated with stem canker of peach, damping off, and root necrosis in fennel and stem rot of coriander [49,50,51,52]. It has also been reported to be a mycoparasite of powdery mildew [85]. *Didymella glomerata* as *P*. *glomerata* has been recorded as an endophytic fungus from Korean pine (*Pinus koraiensis*) leaves [86]. It has also been associated with the pea “Ascochyta blight complex” in Australia [87]. In this study, we first isolated *D. glomerata* from twig spot and gummosis trunk samples from peach worldwide.

*Botryosphaeriaceae* harbours a collection of fungi that exhibit considerable diversity in terms of morphology; these fungi include endophytic, pathogenic, and saprobic variants that primarily affect woody plants. The frequency with which these fungi are involved with plant diseases is substantial. Most species of *Botryosphaeria* are considered latent plant pathogens that cause dieback, cankers, gummosis, leaf spots, or fruit rot on many woody plants, including pear, grape, mango, olive, eucalyptus, maple, oak, and almond [57]. These species are important pathogens of peach and are associated with a series of diseases, including gummosis [59,60,61] and shoot blight [63]. *Botryosphaeria dothidea* is one of the most common species of *Botryosphaeriaceae* and has been reported in hundreds of plant species worldwide [57]. According to a previous study, *B. dothidea* causes perennial cankers in peach tree trunks, branches, and shoots [57]. Gummosis is a common disease of peach that was first observed in the 1970s in Fort Valley, GA, and the causal agent was first identified as *B. dothidea* [59]. A subsequent report revealed that *B. dothidea*, *B. rhodina*, and *B. obtusa* cause peach tree gummosis in Georgia [60]. Chen [61] first reported the occurrence of *B. dothidea* causing gummosis of peach trees in China. Wang reported that *Lasiodiplodia theobromae* or *Diplodia seriata* also cause peach tree gummosis in China [62]. In the present study, we isolated *B. dothidea* from samples collected from four provinces in China, which presented branch canker and gummosis trunk symptoms.

*Diplodia* is a common pathogen on a wide range of hosts. *Diplodia seriata* is associated with olive plants in Tunisia [69] and Croatia [70], and in Uruguay, it was isolated from grapevine [71], apple [72], and peach [88]. *Diplodia mutila* was reported as a new record for olive in Uruguay. Previously, this species was isolated only from pear [89]. In the present study, we isolated *D. seriata* from peach twig spot samples. *Neofusicoccum occulatum* was reported as the pathogen causing shoot blight in *Platycladus orientalis* [74]. Ma et al. [90] first reported that *N. occulatum* was associated with *Dendrobium chrysanthum*. *Neofusicoccum occulatum* was reported as a new record on olives worldwide [89]. It was also described in Australia to affect *Eucalyptus* and *Wollemia nobilis* [91] and subsequently found in blueberry [92]. In this study, we isolated *N. occulatum* from twig canker of peach. *Phaeobotryon* was introduced by Theissen & Sydow [75]. *Phaeobotryon rhois* was previously reported as a pathogen associated with cankers on *Rhus typhinain* in northwestern China [76]. Zhu et al. reported this species from peach on Mount Dongling, Beijing, China [93]. In addition, *Phaeobotryon rhois* has been reported from various hosts including *Dioscoreanipponica*, *Platycladus orientalis*, and *Rhamnus davurica* [94]. In this study, we extended its host range to peach.

*Lasiodiplodia* is commonly associated with diseases of agricultural and forestry crops and has a wide global distribution. This genus is typified by *L. theobromae.* In the present study, we introduce a novel species closely related to *L. acacia.* Zhang et al. [67] reported *L. acacie*, which was isolated for the first time from *Acacia sp*. Identification and characterization of *Lasiodiplodia* species have become challenging recently. Previous studies have mentioned that morphology and phylogeny play an important role in species delineation [37,77]. In addition, some studies have proposed that *Lasiodiplodia* species might have host specificity as well [77]. However, pathogenicity assays are required to understand the relationship of the novel species with the gummosis disease in peach.

*Phaeoacremonium* is associated with stunted growth and dieback in various woody hosts. Damm et al. [82] isolated *Phaeoacremonium* species from necrotic woody tissue of *Prunus* spp. (plum, peach, nectarine, and apricot) from the growing areas of different stone fruits in South Africa. In this study, we isolated *P. scolyti* from peach with gummosis trunk disease.

Based on the results above, it is evident that peach trunk disease is a complex disease that might be caused by different fungal species. These fungal taxa may have varying impacts on peach. However, future studies are required to understand the infection mechanisms and co-infection of these species that lead to peach trunk disease in China. A similar observation has been reported for grapevine woody pathogens. Kraus [95] reported that grapevine wood is a highly complex habitat, with the simultaneous presence of plant pathogens and beneficial, potentially protective fungi. Pathogenic fungi can shift their biotrophic mode from pathogenic to saprotrophic and can become active again under favourable conditions, thus serving as the primary source of inoculation within a vineyard [96]. Therefore, it is important to conduct pathogenicity tests to discern the roles of saprotrophs, endophytes, and pathogens and to investigate the interactions between different communities. Rather than focusing on a single peach branch disease, our research included a comprehensive examination of the various fungi responsible for causing the main symptoms of peach branch diseases throughout China in recent years. More comprehensive field investigations and pathogenicity tests will be implemented in the future.

Overall, early detection and development of management strategies for the correct species identification are important in plant pathology [97]. The present study allowed us to gain a better understanding of the fungal communities associated with peach branch diseases and their roles in the ecosystem. Our results revealed that there was a high diversity of fungi associated with peach branch diseases, with each disease type being dominated by a specific set of fungal species. Additionally, our study revealed that some fungi were present in multiple disease types, suggesting that they may play a role in the progression of multiple diseases. These findings highlight the intricate and complex nature of fungal communities associated with peach branch diseases and emphasize the need for further investigation to fully understand the ecological roles and interactions of fungal communities in peach orchard ecosystems.

## Figures and Tables

**Figure 7:**
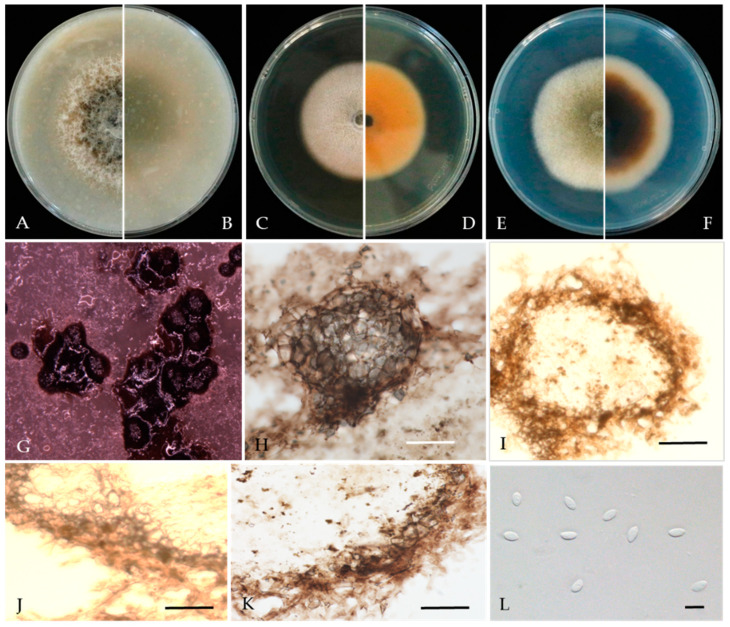
***Nothophoma pruni*** (JZB380123) (**A**,**B**) Colony on OA (front and reverse) (**C**,**D**) Colony on MEA (front and reverse) (**E**,**F**) Colony on PDA (front and reverse) (**G**) Pycnidia forming on OA. (**H**) Pycnidium. (**I**) Section through the pycnidium. (**J**–**K**) Section of the pycnidial wall. (**L**) Conidia. Scale bars: (**H**) = 100 μm; (**I**–**K**) = 50 μm; and (**L**) = 5 μm.

**Figure 8:**
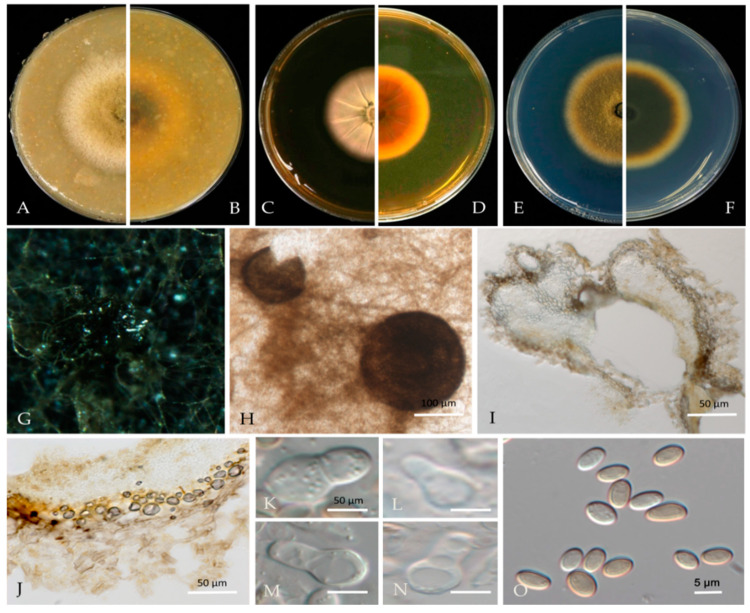
***Nothophoma quercina*** (JZB380133) (**A**,**B**) Colony on OA (front and reverse) (**C**,**D**) Colony on MEA (front and reverse) (**E**,**F**) Colony on PDA (front and reverse) (**G**) Pycnidia forming on OA. (**H**) Pycnidium. (**I**) Section through the pycnidium. (**J**) Section of the pycnidial wall. (**K**–**N**) Conidiogenous cells. (**O**) Conidia. Scale bars: (**H**) = 100 μm; (**I**–**N**) = 50 μm; and (**O**) = 5 μm.

**Figure 12:**
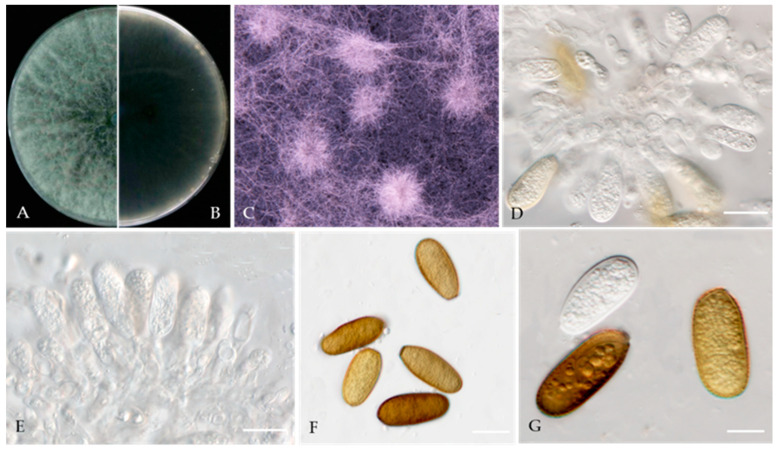
***Diplodia seriata*** (JZB310241) (**A**,**B**) Colony on PDA (front and reverse); (**C**) Appearance of conidiomata on PDA; (**D**,**E**) Conidiogenous cells; (**F**,**G**) Conidia; Scale bars: (**D**–**F**) = 10 μm and (**G**) = 5 μm.

## Data Availability

All sequence data are available in NCBI GenBank following the accession numbers in the manuscript.

## Supplementary Materials

The following supporting information can be downloaded at: https://www.mdpi.com/article/10.3390/jof10030217/s1, Table S1: Strains used in phylogenetic analyses and their GenBank accession numbers.
