# Supplementary material for: Microfungi Associated with Peach Branch Diseases in China
Source: J Fungi (Basel). 2024 Mar 15;10(3):217. doi: 10.3390/jof10030217 (PMC10971364; doi:10.3390/jof10030217)
Supplement: Supplementary file 1 [file jof-10-00217-s001.zip › Supplementary Table 1. Strains used in phylogenetic analyses and their GenBank accession numbers.pdf]

Supplementary Table 1 Strains used in phylogenetic analyses and their GenBank accession numbers.

Species	Isolate	GenBank accession numbers					
		ITS	*tef1*	*tub2*	*rpb2*	LSU	*act*
Ascochyta astragalina	**CBS 113797T**	MH862944		KT389776		MH874509	
A. benningiorum	**CBS 144957T**	MN823432		MN824755	MN824606	MN823432	
A. boeremae	**CBS 372.84T**	KT389480		KT389774		KT389697	
A. boeremae	CBS 373.84	KT389481		KT389775	KT389560	KT389698	
A. clinopodiicola	CBS 123526	MT005692		MT005692		MN943792	
A. fabae	CBS 524.77	GU237880		GU237526		GU237963	
A. fabae	CBS 649.71	GU237902		GU237527		GU237964	
A. herbicola	CBS 629.97	GU237898		GU237614	KP330421	GU238083	
A. lentis	CBS 370.84	KT389474		KT389768		KT389691	
A. medicaginicola var.	BRIP 45051	KY742044		KY742286	KY742132	KY742198	
A. medicaginicola var.	CBS 404.65	GU237859		GU237629	KP330423	GU238102	
A. nigripycnidia	**CBS 116.96T**	GU237756		GU237637		GU238118	
A. phacae	**CBS 184.55T**	KT389475		KT389769		KT389692	
A. pisi	CBS 122751	KP330432		KP330388	EU874867	KP330444	
A. pisi	CBS 12654	GU237772		GU237531	DQ677967	EU754137	
A. prunus	JZB380109	OP753589		OP998335	OP966808	OP753661	
A. prunus	JZB380110	OP753590		OP998336	OP966809	OP753662	
A. prunus	JZB380111	OP753591		OP998337	OP966810	OP753663	
A. prunus	JZB380112	OP753592		OP998338		OP753664	
A. prunus	JZB380113	OP753593			OP966811	OP753665	
A. prunus	JZB380114	OP753594		OP998339		OP753666	
A. prunus	JZB380115	OP753595		OP998340	OP966812	OP753667	
A. prunus	JZB380116	OP753596		OP998341	OP966813	OP753668	

Species	Isolate	GenBank accession numbers					
		ITS	*tef1*	*tub2*	*rpb2*	LSU	*act*
A. rabiei	CBS 206.30	KT389478		KT389772	KT389559	KT389695	
A. rabiei	**CBS 237.37T**	KT389479		KT389773		KT389696	
A. syringae	CBS 545.72	KT389483		KT389777		KT389700	
A. viciae	CBS 451.68	KT389484		KT389778	KT389562	KT389701	
A. viciae-pannonicae	CBS 254.92	KT389485		KT389779		KT389702	
Didymella aeria	CGMCC 3.18353	KY742051		KY742293	KY742137	KY742205	
D. aliena	CBS 379.93	GU237851		GU237578	KP330416	GU238037	
D. aliena	CBS 378.93	MN973539		MT005642	MT018169	MN943746	
D. aliena	CBS 877.97	GU237910		GU237579	MT018168		
D. americana	CBS 185.85	FJ426972		FJ427088	KT389594	GU237990	
D. americana	LC 5157	KY742054		KY742296	KY742139	KY742208	
D. americana	CBS 120140	MN973532		MT005635	MT018155	MN943739	
D. anserina	CBS 167.42	MH856110			MT018152	MH867609	
D. anserina	CBS 397.65	MH85863		KT38979	KT389597	MH870273	
D. anserina	CBS 285.29	MH855074		KT389796	MT018151	MH866534	
D. aurea	**CBS 269.93T**	GU237818		GU237557	KT389599	GU237999	
D. gardeniae	**CBS 626.68T**	FJ427003		FJ427114	KT389606	GQ387595	
D. gardeniae	CBS 302.79	FJ427002		FJ427113	MT018156		
D. glomerata	LC 8124	KY742063		KY742305	KY742148	KY742217	
D. glomerata	CBS 128899	MH865146			MT018147	MH876585	
D. glomerata	JZB380117	OP753597		OQ032564	OP966814	OP753669	
D. glomerata	JZB380118	OP753598		OQ032565	OP966815	OP753670	
D. glomerata	JZB380119	OP753599		OQ032566	OP966816	OP753671	
D. glomerata	JZB380120	OP753600		OQ032567	OP966817	OP753672	

Species	Isolate	GenBank accession numbers					
		ITS	*tef1*	*tub2*	*rpb2*	LSU	*act*
D. glomerata	JZB380121	OP753601		OQ032568	OP966818	OP753673	
D. glomerata	JZB380122	OP753602		OQ032569	OP966819	OP753674	
D. heteroderae	**CBS 109.92T**	FJ426983		FJ427098	KT389601	GU238002	
D. heteroderae	LC 8125	KY742064		KY742306	KY742149	KY742218	
D. longicolla	CBS 347 82	GU237836		GU237621	MT018160		
D. longicolla	**CBS 124514T**	MH863382		GU237622	MT018161		
D. nigricans	CBS 444.81	GU237867		GU237558		GU238000	
D. nigricans	PD 77/919	GU237915		GU237559	KT389611	GU238001	
D. prosopidis	CBS 136414			MT005631	MT018149		
D. prosopidis	CBS 136550	MH866094			MT018150	MH877632	
D. protuberans	**CBS 381.96T**	GU237853		GU237574	KT389620	GU238029	
D. protuberans	CBS 391.93	GU237858		GU237566	KT389621	GU238015	
D. sinensis	CGMCC 3.18348	KY742085		KY742327	MT018127	KY742239	
Nothophoma anigozanthi	**CBS 381.91T**	GU237852		GU237580	KT389655	GU238039	
N. gossypiicola	CBS 377.67	GU237845		GU237611	KT389658	GU238079	
N. macrospora	**CBS 140674T**	NR158272		LN880539			
N. pruni	JZB380123	OP753603		OQ032570	OP998319	OP753675	
N. pruni	JZB380124	OP753604		OQ032571		OP753676	
N. pruni	JZB380125	OP753605		OQ032572	OP998320	OP753677	
N. pruni	JZB380126	OP753606		OQ032573	OP998321	OP753678	
N. pruni	JZB380127	OP753607		OQ032574	OP998322	OP753679	
N. pruni	JZB380128	OP753608		OQ032575		OP753680	
N. pruni	JZB380129	OP753609		OQ032576	OP998323	OP753681	
N. pruni	JZB380130	OP753610		OQ032577	OP998324	OP753682	

Species	Isolate	GenBank accession numbers					
		ITS	*tef1*	*tub2*	*rpb2*	LSU	*act*
N. pruni	JZB380131	OP753611		OQ032578		OP753683	
N. pruni	JZB380132	OP753612		OQ032579	OP998325	OP753684	
N. pruni	JZB380135	OP753613		OQ032580	OP998327	OP753685	
N. pruni	MFLUCC 18-1601	MH827005		MH853669	MH853662	MH853662	
N. pruni	MFLUCC 18-1600	MH827007		MH853671	MH853664	MH827028	
N. quercina	CBS 633.92	GU237900		GU237609	KT389657	EU754127	
N. quercina	UTHSC DI16-270	LT592929		LT592998		LT593067	
N. quercina	JZB380133	OP753614				OP753686	
N. quercina	JZB380134	OP753615		OQ032581	OP998326	OP753687	
N. quercina	JZB380136	OP753616		OQ032582	OP998328	OP753688	
N. quercina	JZB380137	OP753617		OQ032583	OP998329	OP753689	
N. quercina	JZB380138	OP753618		OQ032584	OP998330	OP753690	
N. quercina	JZB380139	OP753619		OQ032585		OP753691	
N. quercina	JZB380140	OP753620		OQ032586	OP998331	OP753692	
N. quercina	JZB380141	OP753621		OQ032587	OP998332	OP753693	
N. quercina	JZB380142	OP753622		OQ032588	OP998333	OP753694	
N. quercina	JZB380143	OP753623				OP753695	
N. quercina	JZB380144	OP753624		OQ032589	OP998334	OP753696	
N. quercina	MFLUCC 18-1588	MH827008		MH853672	MH853665	MH827029	
N variabilis	UTHSC DI16-285	NR158280		LT593008	LT593008	LT593078	
Phaeoacremonium alvesii	CSN1239			KY906785			KY906784
P. alvesii	CSN1335			*KY906801*			*KY906800*
P. aureum	CBS 142690			KY906799			KY906798
P. aureum	**CBS 142691T**			KY906657			KY906656

Species	Isolate	GenBank accession numbers					
		ITS	*tef1*	*tub2*	*rpb2*	LSU	*act*
P. australiense	CSN490			KY906729			KY906728
P. australiense	CSN657			KY906735			KY906734
P. griseorubrum	PMM1828			KY906851			KY906850
P. griseorubrum	PMM1895			KY906875			KY906874
P. italicum	CSN59			KY906677			KY906676
P. italicum	CSN119			KY906691			KY906690
P. junior	**CBS 142695T**			KY906651			KY906650
P. junior	CBS 142696			KY906653			KY906652
P. parasiticum	CSN72			KY906683			KY906682
P. parasiticum	CSN79			KY906687			KY906686
P. paululum	**CBS 142705T**			KY906881			KY906880
P. proliferatum	**CBS 142706T**			KY906903			KY906902
P. proliferatum	CBS 142707			KY906827			KY906826
P. scolyti	CSN27			KY906661			KY906660
P. scolyti	CSN55			KY906671			KY906670
P. scolyti	JZB3190015			OR831988			OR831985
P. scolyti	JZB3190016			OR831989			OR831986
P. scolyti	JZB3190017			OR831990			OR831987
P. subulatum	CSN42			KY906663			KY906662
P. subulatum	CSN51			KY906669			KY906668
P. venezuelense	PMM1138			KY906835			KY906834
Pleurostoma richardsiae	**CBS 270.33T**			AY579334			AY579271
Barriopsis iraniana	CBS 124698	MH863399			KX463949	MH874917	
Botryosphaeria agaves	MFLUCC 10-0051	JX646790	JX646855	JX646840			

Species	Isolate	GenBank accession numbers					
		ITS	*tef1*	*tub2*	*rpb2*	LSU	*act*
B. agaves	**CBS 133992T**	JX646791	JX646856	JX646841			
B. cortices	**CBS 119047T**	DQ299245	EU017539	EU673107			
B. cortices	ATCC 22927	DQ299247	EU673291	EU673108			
B. auasmontanum	**CBS 121769T**	EU101303	EU101348				
B. dothidea	CBS 115476				GU357802	DQ377852	
B. dothidea	CBS 110302	AY259092	AY573218	EU673106			
B. dothidea	CBS 110484	AY236949	AY236898	AY236927			
B. dothidea	CBS 121768	EU101306.	EU101351				
B. dothidea	JZB310244	OP926938	OQ032594	OQ032630			
B. dothidea	JZB310245	OP926939	OQ032595	OQ032631			
B. dothidea	JZB310246	OP926940	OQ032596	OQ032632			
B. dothidea	JZB310247	OP926941	OQ032597	OQ032633			
B. dothidea	JZB310248	OP926942	OQ032598	OQ032634			
B. dothidea	JZB310249	OP926943	OQ032599	OQ032635			
B. dothidea	JZB310250	OP926944	OQ032600	OQ032636			
B. dothidea	JZB310251	OP926945	OQ032601	OQ032637			
B. dothidea	JZB310252	OP926946	OQ032602	OQ032638			
B. dothidea	JZB310253	OP926947	OQ032603	OQ032639			
B. dothidea	JZB310254	OP926948	OQ032604	OQ032640			
B. dothidea	JZB310255	OP926949	OQ032605	OQ032641			
B. dothidea	JZB310256	OP926950	OQ032606	OQ032642			
B. dothidea	JZB310257	OP926951	OQ032607	OQ032643			
B. dothidea	JZB310258	OP926952	OQ032608	OQ032644			
B. dothidea	JZB310259	OP926953	OQ032609	OQ032645			

Species	Isolate	GenBank accession numbers					
		ITS	*tef1*	*tub2*	*rpb2*	LSU	*act*
B. dothidea	JZB310260	OP926954	OQ032610	OQ032646			
B. dothidea	JZB310261	OP926955	OQ032611	OQ032647			
B. dothidea	JZB310262	OP926956	OQ032612	OQ032648			
B. dothidea	JZB310263	OP926957	OQ032613	OQ032649			
B. dothidea	JZB310264	OP926958	OQ032614	OQ032650			
B. dothidea	JZB310265	OP926959					
B. dothidea	JZB310266	OP926960	OQ032615	OQ032651			
B. dothidea	JZB310267	OP926961	OQ032616	OQ032652			
B. dothidea	JZB310268	OP926962	OQ032617	OQ032653			
B. dothidea	JZB310269	OP926963	OQ032618	OQ032654			
B. dothidea	JZB310270	OP926964	OQ032619	OQ032655			
B. dothidea	JZB310271	OP926965	OQ032620	OQ032656			
B. dothidea	JZB310272	OP926966	OQ032621	OQ032657			
B. dothidea	JZB310273	OP926967	OQ032622	OQ032658			
B. dothidea	JZB310274	OP926968	OQ032623				
B. dothidea	JZB310275	OP926969	OQ032624	OQ032659			
B. dothidea	JZB310276	OP926970	OQ032625	OQ032660			
B. dothidea	CBS 116777	AY786320	AY786317				
B. fabicerciana	**CMM27121**	HQ332198	HQ332214	KF779069			
B. fabicerciana	CERC 2006	KX277978	KX278083	KX278188			
B. kuwatsukai	**CBS 135219T**	KX197074	KX197094	KX197101			
B. kuwatsukai	LSP5	KJ433395	KJ433417				
B. minutispermatia	GAAS-01	KX447675	KX447678				
B. minutispermatia	GAAS-02	KX447676	KX447679				

Species	Isolate	GenBank accession numbers					
		ITS	*tef1*	*tub2*	*rpb2*	LSU	*act*
B. qingyuanensis	**CERC 2946T**	KX278000	KX278105	KX278209			
B. qingyuanensis	CERC 2947	KX278001	KX278106	KX278210			
B. ramose	**CBS122069T**	EU144055	EU144070	KF766132			
B. rosaceae	LSP5	KJ433395	KJ433417				
B. scharifii	CBS 124702	JQ772019	JQ772056				
B. scharifii	**CBS 124703T**	JQ772020	JQ772057				
B. sinensis	CGMCC 3.17723	KT343256	KU221234	KX197108			
B. sinensis	CGMCC 3.17724	KU997394	KU997130	KU997568			
B. wangensis	CERC 2299	KT343254	KU221233	KX197107			
B. wangensis	CERC 2298	KX278002	KX278107	KX278211			
Diplodia.africana	**CBS 120835T**	EF445343	EF445382	KF766129			
D. africana	CBS 121104	EF445344	EF445383				
D. allocellula	**CBS 130408T**	JQ239397	JQ239384	JQ239378			
D. allocellula	CBS 130410	JQ239399	JQ239386	JQ239380			
D. bugarica	CBS 124136	MH863355	GQ923822	MT592475			
D. bugarica	**CBS 124135T**	GQ923852	GQ923820	MT592474			
D. citricarpa	CBS 124714	MT587336	MT592039	MT592478			
D. citricarpa	**CBS 124715T**	KF890207	KF890189	KX464784			
D. corticola	CBS 112549	AY259100	AY573227	DQ458853			
D. corticola	CBS 112546	AY259090	EU673310	EU673117			
D. cupressi	CBS 120692	MT587338	MT592043	MT592485			
D. cupressi	**CBS 168.87T**	DQ458893	DQ458878	DQ458861			
D. eriobotryicola	**CBS 140851T**	MT587342	MT592047	MT592489			
D. eriobotryicola	CPC 29680	KT240355	KT240193	MG015806			

Species	Isolate	GenBank accession numbers					
		ITS	*tef1*	*tub2*	*rpb2*	LSU	*act*
D. estuarina	**CBS 139666T**	KP860831	KP860676	KP860754			
D. estuarina	CBS 139668	MT587344	MT592049	MT592491			
D. fraxin	**CBS 136010T**	KF307700	KF318747	MG015807			
D. fraxin	CBS 136011	KF307711	KF318758	MG015808			
D. mutila	**CBS 136014T**	KJ361837	KJ361829	MG015815			
D. mutila	CBS 136017	KJ361840	KJ361832	MT592514			
D. mutila	CMW 7060	AY236955	AY236904	AY236933			
D. pseudoseniata	**CBS 124906T**	EU080927	EU863181	MG015820			
D. pseudoseniata	CBS 125527	MH863604	MT592059	MT592501			
D. sapinea	**CBS 393.84T**	DQ458895	DQ458880	DQ458863			
D. sapinea	CBS 591.84	MT587367	MT592073	MT592530			
D. scrobiculata	CBS 113297	KX464102	AY624254	AY624259			
D. scrobiculata	**CBS 118110T**	AY253292	AY624253	AY624258			
D. seriata	JZB310240	OP926934	OQ032590	OQ032626			
D. seriata	JZB310241	OP926935	OQ032591	OQ032627			
D. seriata	JZB310242	OP926936	OQ032592	OQ032628			
D. seriata	JZB310243	OP926937	OQ032593	OQ032629			
D. seriata	**CBS 112555T**	AY259094	AY573220	DQ458856			
D. seriata	CBS 124137	MT587384	GQ923845	MT592559			
D. seriata	CBS 215.25	MT587388	MT592099	MT592568			
D. seriata	CBS 585.50	MT587389	MT592100	MT592569			
Neofusicoccum arbuti	CBS 116131	AY819720	KF531792	KF531793			
N. arbuti	CBS 117090	AY819724	KF531791	KF531794			
N. brasiliense	CMM1285	JX513628	JX513608	KC794030			

Species	Isolate	GenBank accession numbers					
		ITS	*tef1*	*tub2*	*rpb2*	LSU	*act*
N. brasiliense	**CMM1338T**	JX513630	JX513610	KC794031			
N. cordaticola	**CBS 123634T**	EU821898	EU821868	EU821838			
N. cordaticola	CBS 123635	EU821903	EU821873	EU821843			
N. illicii	CGMCC 3.18311	KY350150	KY817756	KY350156			
N. illicii	**CGMCC 3.18310T**	KY350149		KY350155			
N. kwambonambiense	**CBS 123639T**	EU821900	EU821870	EU821840			
N. kwambonambiense	CBS 123641	EU821919	EU821889	EU821859			
N. macroclavatum	**CBS 118223T**	DQ093196	DQ093217	DQ093206			
N. nonquaesitum	**CBS 126655T**	GU251163	GU251295	GU251823			
N. nonquaesitum	CBS 133501	MT587498	MT592213	MT592705			
N. nonquaesitum	PD301	GU251164	GU251296	GU251824			
N. occulatum	**CBS 128008T**	EU301030	EU339509	EU339472			
N. occulatum	CBS 519.74	MT587499	MT592214	MT592706			
N. occulatum	JZB3120010	OR449404	OR514830	OR514833			
N. occulatum	JZB3120011	OR449405	OR514831	OR514834			
N. occulatum	JZB3120012	OR449406	OR514832	OR514835			
N. ribis	CBS 114306	MT587514	MT592229	MT592721			
N. ribis	**CBS 115475T**	AY236935	AY236877	AY236906			
N. sinense	**CGMCC 3.18315T**	KY350148	KY817755	KY350154			
N. sinoeucalypti	**CGMCC 3.18752T**	KX278061	KX278166	KX278270			
N. sinoeucalypti	CERC 3415	KX278063	KX278168	KX278272			
Phaeobotryon aplospora	CFCC 53774	MN215836	MN205996			MN215871	
P. aplospora	**CFCC 53775T**	MN215837				MN215872	
P. aplospora	CFCC 53776	MN215838	MN205997			MN215873	

Species	Isolate	GenBank accession numbers					
		ITS	*tef1*	*tub2*	*rpb2*	LSU	*act*
P. mamane	CPC 12442	EU673333	EU673299			DQ377899	
P. mamane	**CBS 122980T**	KF766209	EU673298			EU673248	
P. mamane	CPC 12443	EU673334	EU673300			EU673249	
P. negundinis	CAA797	KX061513	KX061507				
P. negundinis	CAA798	KX061514	KX061508				
P. rhoinum	CFCC 52449	MH133923	MH133957			MH133940	
P. rhoinum	**CFCC 52450T**	MH133924	MH133958			MH133941	
P. rhoinum	CFCC 52451	MH133925	MH133959			MH133942	
P. rhois	CFCC 89662	KM030584	KM030598			KM030591	
P. rhois	**CFCC 52448T**	MH133922	MH133956			MH133939	
P. rhois	JZB3600007	OR431839	OR514836			OR482137	
P. rhois	JZB3600008	OR431840				OR482138	
P. rhois	JZB3600009	OR431841	OR514837			OR482139	
P. ulmi	**CBS 138854T**	MT587540	MT592274			MT587321	
P. ulmi	CBS 114123	MT587539	MT592273			MT587320	
P. cupressi	IRAN1454C	FJ919673	FJ919662				
P. cupressi	IRAN 1445C	KF766208	KF766428			KF766295	
P. cupressi	CBS 124700T	MH863400					
Lasiodiplodia acaciae	**CBS 136434T**	MT587421	MT592133	MT592613			
L. aquilariae	**CGMCC 3.18471T**	KY783442	KY848600	–			
L. avicenniae	**CBS 139670T**	KP860835	KP860680	KP860758			
L. avicenniae	**LAS 199**	KU587957	KU587947	KU587868			
L. brasiliensis	**CMM 4015T**	JX464063	JX464049	–			
L. brasiliensis	CMM 4469	KT325574	KT325580	–			

Species	Isolate	GenBank accession numbers					
		ITS	*tef1*	*tub2*	*rpb2*	LSU	*act*
L. bruguierae	**CBS 139669T**	KP860832	KP860677	KP860755			
L. bruguierae	CBS 141453	KP860834	KP860679	KP860757			
L. caatinguensis	**CMM 1325T**	KT154760	KT008006	KT154767			
L. caatinguensis	IBL 381	KT154757	KT154751	KT154764			
L. chiangraiensis	MFLU 21-0003	MW760854	MW815630	MW815628			
L. chiangraiensis	GZAAS 21-0003	MW760853	MW815629	MW815627			
L. chonburiensis	PSU-LST002	LC762194	LC762200	LC762206			
L. chonburiensis	PSU-LST003	LC762195	LC762201	LC762207			
L. cinnamomi	**CFCC 51997T**	MG866028	MH236799	MH236797			
L. cinnamomi	CFCC 51998	MG866029	MH236800	MH236798			
L. citricola	CBS 124706	GU945353	GU945339	KU887504			
L. citricola	**CBS 124707T**	GU945354	GU945340	KU887505			
L. crassispora	**CBS 118741T**	DQ103550	DQ103557	KU887506			
L. crassispora	CMW 13488	DQ103552	DQ103559	KU887507			
L. curvata	**CGMCC 3.18456T**	KY783437	KY848596	KY848529			
L. curvata	CGMCC 3.18476	KY783443	KY848601	KY848532			
L laeliocattleyae	**CBS 130992T**	JN814397	JN814424	KU887508			
L. laeliocattleyae	**BOT 29**	JN814401		JN814428			
L. euphorbiaceicola	**CMM 3609T**	KF234543	KF226689	KF254926			
L. euphorbiaceicola	CMW 33268	KU887131	KU887008	KU887430			
L. exigua	BL184	KJ638318	KJ638337	–			
L. exigua	**CBS 137785T**	KJ638317	KJ638336	KU887509			
L. gilanensis	**CBS 124704T**	GU945351	GU945342	KU887511			
L. gilanensis	CBS 124705	GU945352	GU945341	KU887510			

Species	Isolate	GenBank accession numbers					
		ITS	*tef1*	*tub2*	*rpb2*	LSU	*act*
L. gonubiensis	**CMW 14077T**	AY639595	DQ103566	DQ458860			
L. gonubiensis	CMW 14078	AY639594	DQ103567	EU673126			
L. gravistriata	**CMM 4564T**	KT250949	KT250950	–			
L. gravistriata	CMM 4565	KT250947	KT266812	–			
L. hormozganensis	**CBS 124709T**	GU945355	GU945343	KU887515			
L. hormozganensis	CBS 124708	GU945356	GU945344	KU887514			
L. iraniensis	**CBS 124710T**	GU945348	GU945336	KU887516			
L. iraniensis	CBS 124711	GU945347	GU945335	KU887517			
L. laosensis	**CGMCC 3.18464T**	KY783471	KY848609	KY848552			
L. laosensis	CGMCC 3.18473	KY783450	KY848603	KY848536			
L. lignicola	**CBS 134112T**	JX646797	KU887003	JX646845			
L. lignicola	**CGMCC 3.18061T**	KX499889	KX499927	KX500002			
L. macrospora	**CMM 3833T**	KF234557	KF226718	KF254941			
L. mahajangana	**CBS 124925T**	FJ900595	FJ900641	FJ900630			
L. mahajangana	CBS 124926	FJ900596	FJ900642	FJ900631			
L. margaritacea	CBS 122065	EU144051	EU144066	–			
L. margaritacea	**CBS122519T**	EU144050	EU144065				
L. mediterranea	**CBS 137783T**	KJ638312	KJ638331	KU887521			
L. mediterranea	CBS 137784	KJ638311	KJ638330	KU887522			
L. microconidia	**CGMCC 3.18485T**	KY783441	KY848614	–			
L. parva	**CBS 456.78T**	EF622083	EF622063	KU887523			
L. parva	CBS 494.78	EF622084	EF622064	EU673114			
L. plurivora	CBS 121103	AY343482	EF445396	KU887525			
L. plurivora	**STE-U 5803T**	EF445362	EF445395	KP872421			

Species	Isolate	GenBank accession numbers					
		ITS	*tef1*	*tub2*	*rpb2*	LSU	*act*
L. pontae	**CMM 1277T**	KT151794	KT151791	KT151797			
L. ponkanicola	**CGMCC3.20388**	MW880685	MW884188	MW884214			
L. prunus	JZB3130029	OR821993	OR831982	OR831991			
L. prunus	JZB3130030	OR821994	OR831983	OR831992			
L. prunus	JZB3130031	OR821995	OR831984	OR831993			
L. pseudotheobromae	**CBS 116459T**	EF622077	EF622057	EU673111			
L. pseudotheobromae	CBS 116460	EF622078	EF622058	KU198428			
L. pyriformis	**CBS 121770T**	EU101307	EU101352	KU887527			
L. pyriformis	CBS 121771	EU101308	EU101353	KU887528			
L. rubropurpurea	**CBS 118739T**	DQ103553	DQ103571	EU673136			
L. rubropurpurea	WAC 12536	DQ103554	DQ103572	KU887530			
L. subglobosa	**CMM 3872T**	KF234558	KF226721	KF254942			
L. subglobosa	CMM 4046	KF234560	KF226723	KF254944			
L. syzygii	MFLUCC 19.0257	–	MW016943	MW014331			
L. thailandica	CBS 138653	KM006433	KM006464	–			
L. thailandica	**CBS 138760T**	KJ193637	KJ193681	–			
L. theobromae	CBS 111530	EF622074	EF622054	KU887531			
L. theobromae	**CBS 164.96T**	AY640255	AY640258	KU887532			
L. tropica	CGMCC 3.18477	KY783454	KY848616	KY848540			
L. vaccinii	**CGMCC 3.19022T**	MH330318	MH330327	MH330324			
L. venezuelensis	**CBS 118739T**	DQ103547	DQ103568	KU887533			
L. venezuelensis	CMW 13512	DQ103548	DQ103569	KU887534			
L. viticola	**CBS 128313T**	HQ288227	HQ288269	HQ288306			
L. viticola	CBS 128314	HQ288228	HQ288270	HQ288307			

Species	Isolate	GenBank accession numbers					
		ITS	*tef1*	*tub2*	*rpb2*	LSU	*act*
L. vitis	**CBS 124060T**	KX464148	MN938928	KX464917			

CBS: Culture collection of the Centraalbureau voor Schimmelcultures, Fungal Biodiversity Centre, Utrecht, The Netherlands; CPC: Personal collection of P.W. Crous, Utrecht, The Netherlands;

JZB: Beijing Academy of Agriculture and Forestry Sciences culture collection, Beijing, China;

MFLUCC: Mae Fah Luang University Culture Collection, Thailand;

ITS: internal transcribed spacer regions 1 & 2 including 5.8S nrDNA gene; *tub2*: beta-tubulin; *rpb*2: RNA polymerase II second subunit; *tef1*: Partial translation elongation factor 1-α; LSU: Ribosomal 28S RNA; *act*: actin.
